# Covariance Between Genotypic Effects and its Use for Genomic Inference in Half-Sib Families

**DOI:** 10.1534/g3.116.032409

**Published:** 2016-07-07

**Authors:** Dörte Wittenburg, Friedrich Teuscher, Jan Klosa, Norbert Reinsch

**Affiliations:** Leibniz Institute for Farm Animal Biology, Institute of Genetics and Biometry, 18196 Dummerstorf, Germany

**Keywords:** autoregressive prior, Bayesian statistics, linkage disequilibrium, recombination rate, SNP effect

## Abstract

In livestock, current statistical approaches utilize extensive molecular data, *e.g.*, single nucleotide polymorphisms (SNPs), to improve the genetic evaluation of individuals. The number of model parameters increases with the number of SNPs, so the multicollinearity between covariates can affect the results obtained using whole genome regression methods. In this study, dependencies between SNPs due to linkage and linkage disequilibrium among the chromosome segments were explicitly considered in methods used to estimate the effects of SNPs. The population structure affects the extent of such dependencies, so the covariance among SNP genotypes was derived for half-sib families, which are typical in livestock populations. Conditional on the SNP haplotypes of the common parent (sire), the theoretical covariance was determined using the haplotype frequencies of the population from which the individual parent (dam) was derived. The resulting covariance matrix was included in a statistical model for a trait of interest, and this covariance matrix was then used to specify prior assumptions for SNP effects in a Bayesian framework. The approach was applied to one family in simulated scenarios (few and many quantitative trait loci) and using semireal data obtained from dairy cattle to identify genome segments that affect performance traits, as well as to investigate the impact on predictive ability. Compared with a method that does not explicitly consider any of the relationship among predictor variables, the accuracy of genetic value prediction was improved by 10–22%. The results show that the inclusion of dependence is particularly important for genomic inference based on small sample sizes.

In whole-genome regression analyses, it is often the case that the number of genomic markers, *p*, exceeds that of the observations, *n*. Moreover, linkage and linkage disequilibrium (LD) between loci adds a second source of dependency among the predictors. The number of genomic markers such as single nucleotide polymorphisms (SNPs) is still growing, *e.g.*, ∼26 million SNPs have been identified in the whole-genome sequences of cattle (Daetwyler *et al.* 2014). Genomic prediction works reasonably well when based on a huge number of explanatory variables (*e.g.*, Gianola 2013), but the high dependency among predictors, which is often called multicollinearity, may lead to the incorrect genomic inference of marker effects because the standard error of the estimated effects is likely to be high.

For “p>n” problems, various methods are available that implicitly consider dependencies by selecting relevant predictors and/or shrinking the effect sizes (for a thorough review, see de los Campos *et al.* 2013). Bayesian (*e.g.*, Meuwissen *et al.* 2001; Habier *et al.* 2011) and penalized (*e.g.*, Gianola *et al.* 2006; Piepho 2009) methods are the most common choices for genomic prediction. However, explicitly exploiting dependencies, especially those due to the proximity of SNPs, relies on the appropriate order of loci. Using the order of SNPs, and clustering them according to their adjacency combined with the Group Lasso method can obtain better performance than other penalized approaches, where clusters that contain causal variants may be identified with more confidence (Dehman *et al.* 2015). Alternatively, haplotype-based approaches (*e.g.*, Calus *et al.* 2008; Cuyabano *et al.* 2014) exploit the connections between SNPs, which may improve the accuracy of genetic value prediction. Associations throughout the genome can also be modeled using a first-order antedependence correlation structure (Yang and Tempelman 2012). A penalty term placed on successive differences between the coefficients is employed to consider the natural order of effects, thereby yielding estimates with smooth transitions. In addition, sparsity in terms of nonzero effect estimates can be achieved by penalizing the $L_{1}$ norm of differences in the fused lasso approach (Tibshirani *et al.* 2005). In a Bayesian smoothing framework, random-walk priors were suggested by Fahrmeir and Lang (2001), which also allow nonequal distances between predictors. In particular, for genetic applications, Gianola *et al.* (2003) described an autoregressive process with variable distances between markers in a mixed-model formulation. Incorporating the dependencies among SNPs can improve the outcomes of genomic evaluations (Yang and Tempelman 2012), but the assumed pattern of the covariance structure has been based on general assumptions in previous studies.

The objective of the present study was to extend the Bayesian approach by using an autoregressive prior for marker effects, and to determine the autocorrelation parameter explicitly according to genetic theory. The population structure influences the extent of associations. Family stratification leads to different levels of LD among families, and may result in a biased population-wide measure of LD. Thus, dependencies caused by LD were theoretically derived for a single half-sib family that is part of a typical livestock population (*e.g.*, dairy cattle). This required the haplotypes and recombination rates of the common parent (sire) as well as the LD of gametes in the population from which the individual parent (dam) was derived. The obtained covariance structure was then integrated into a statistical model for genomic evaluation. Genome segments with significant impacts on a quantitative trait were inferred, and the precision of the parameter estimates as well as the accuracy of genomic prediction were evaluated. This report ends with a discussion of partial successes, drawbacks, and further options for considering dependencies.

## Materials and Methods

### Statistical model

To study the genetic effects captured by SNPs, each with two alleles, A and B, the following whole genome regression model is fitted to a trait $y={\left(y_{1},\ldots ,y_{n}\right)}^{\prime }$,

y=1μ+Xm+e. (1)

The design matrix $X={\left\{X_{i,j}\right\}}_{i,j}$ contains the genotype codes at locus j∈{1,…,p} for individual i∈{1,…,n}, where 1 and −1 indicate homozygous genotypes AA and BB, respectively, and the heterozygote is coded as 0. The vector $m={\left(m_{1},\ldots ,m_{p}\right)}^{\prime }$ contains the additive genotype effect at each SNP (*i.e.*, half the difference between homozygotes). Furthermore, *μ* denotes the overall mean, and **1** is a vector of *n* ones. The residuals are assumed to be independent and normally distributed, $e_{i}∼N\left(0,\sigma_{e}^{2}\right)$ for i=1,…,n. For convenience, other effects are omitted.

### Covariance between SNP genotypes

In this section, model (1) is extended to consider the dependencies among predictor variables in X due to linkage and LD specifically for a paternal half-sib family design. The covariance between genotype codes can be derived theoretically for each pair of SNPs. The derivation is based on Bonk *et al.* (2016), who deduced the covariance between SNP genotypes coding for additive and/or dominance effects in a general mating, where the haplotypes of both parents are available. In the main result for the additive effects of SNP alleles, the covariance can be split into paternal (*s*) and maternal (*d*) contributions because the alleles are inherited independently. In extension to Bonk *et al.* (2016), the maternal contribution must be generalized. It is assumed that a dam is drawn randomly from the population. Half-sibs have a common sire, so the covariance between loci *j* and *k* is determined according to the sire’s diplotype S,

$$K_{j,k}\;:=\text{cov}\left(X_{i,j},X_{i,k}|S\right)=\text{cov}\left(X_{i,j,s},X_{i,k,s}|S\right)+\text{cov}\left(X_{i,j,d},X_{i,k,d}\right),$$ (2)

where $X_{i,j,s}$ and $X_{i,j,d}$ take a value of $\frac{1}{2}$ if the A allele was inherited, but $-\frac{1}{2}$ otherwise, and $X_{i,j}=X_{i,j,s}+X_{i,j,d}$.

To determine the paternal contribution to the covariance in Equation (2), three different types of sire diplotypes are distinguished (the results were taken from Bonk *et al.* 2016).

1. Double homozygous sire (haplotypes AA and AA): all half-sibs inherit the same paternal haplotype AA, and the paternal covariance is zero.
2. The sire is heterozygous at one locus (haplotypes AA and AB): two haplotypes AA and AB can be observed among half-sibs, where each is equally frequent. The paternal covariance is also zero.
3. Double heterozygous sire (haplotypes AA and BB or AB and BA): all possible haplotypes appear among daughters with a probability that depends on the recombination rate $\theta_{j,k}$. Thus, $\text{cov}\left(X_{i,j,s},X_{i,k,s}|AA/BB\right)=\frac{1}{4}\left(1-2\theta_{j,k}\right)$ or $\text{cov}\left(X_{i,j,s},X_{i,k,s}|AB/BA\right)=-\frac{1}{4}\left(1-2\theta_{j,k}\right)$. If j=k, then $\theta_{j,k}=0$.

Using the allele frequency, $p_{j}^{A}$, and haplotype frequencies of the maternal gametes, $p_{j,k}^{XY}$, the second part of Equation (2) is:

$$\text{cov}\left(X_{i,j,d},X_{i,k,d}\right)=E\left(X_{i,j,d}X_{i,k,d}\right)-E\left(X_{i,j,d}\right)E\left(X_{i,k,d}\right),$$

$$E\left(X_{i,j,d}X_{i,k,d}\right)=p_{j,k}^{AA}\frac{1}{4}-p_{j,k}^{AB}\frac{1}{4}-p_{j,k}^{BA}\frac{1}{4}+p_{j,k}^{BB}\frac{1}{4},$$

$$E\left(X_{i,j,d}\right)=p_{j}^{A}-\frac{1}{2},$$

$$E\left(X_{i,k,d}\right)=p_{k}^{A}-\frac{1}{2}.$$

Combining the terms yields $\text{cov}\left(X_{i,j,d},X_{i,k,d}\right)=p_{j,k}^{AA}p_{j,k}^{BB}-p_{j,k}^{AB}p_{j,k}^{BA}=D_{j,k}$, the LD of maternal gametes. If j=k, then $\text{cov}\left(X_{i,j,d},X_{i,j,d}\right)=\text{var}\left(X_{i,j,d}\right)=p_{j}^{A}\left(1-p_{j}^{A}\right)$.

In summary, the covariance between SNP genotypes among half-sibs can be split into a linkage part contributed by the sire and an LD part added by the mother. The covariance matrix $K={\left\{K_{j,k}\right\}}_{j,k=1}^{p}$ is set up with the following elements:

$$K_{j,k}=\{\begin{array}{ll} D_{j,k}+\frac{1}{4}\left(1-2\theta_{j,k}\right), & \text{for}\;\text{sire}\;\text{with}\;\text{haplotypes}\;\text{AA}\;\text{and}\;\text{BB}\\ D_{j,k}-\frac{1}{4}\left(1-2\theta_{j,k}\right), & \text{for}\;\text{sire}\;\text{with}\;\text{haplotypes}\;\text{AB}\;\text{and}\;\text{BA}\\ D_{j,k}, & \text{else}. \end{array}$$

The linkage phase of sire, the corresponding recombination rate ($\theta_{j,k}$), and the LD ($D_{j,k}$) of maternal gametes are assumed to be known. If they are not available, these population parameters may be estimated, such as using the maximum likelihood approach (Gomez-Raya *et al.* 2013). Now it is necessary to determine whether genomic evaluations can be improved by knowing this covariance structure.

### Specification of prior assumptions

To estimate the unknown parameters of model (1), a Bayesian shrinkage approach is employed. The matrix K can be incorporated as a scale matrix during the specification of the prior assumptions. Thus, a hierarchical structure is defined for model (1):

$$y|\mu ,m,\sigma_{e}^{2}∼N\left(1\mu +\mathrm{Xm},I\sigma_{e}^{2}\right),$$

m|Ψ∼N(0,Ψ),

μ∝constant,

$$\sigma_{e}^{2}∼\chi^{-2}\left(-2,0\right),$$

where $\chi^{-2}\left(\nu ,S\right)$ denotes the inverse $\chi^{2}$ distribution with *ν* degrees of freedom and scaling parameter *S*. Let Ψ=Ψ(K) be a covariance matrix that depends on the elements of K.

The SNP effects can be estimated as the mean of their posterior distribution, which can be obtained by Gibbs sampling from the conditional distribution (*e.g.*, Sorensen and Gianola 2002)

$$m|y,\Psi ,\mu ,\sigma_{e}^{2}\propto N\left(\Sigma X^{\prime }\left(y-1\mu \right),\Sigma \sigma_{e}^{2}\right)\;\text{with}\;\Sigma =\;{\left(X^{\prime }X+\Psi^{-1}\sigma_{e}^{2}\right)}^{-1}.$$ (3)

Several specifications of Ψ are suitable, which differ in terms of the dynamics of their regularization parameters, as follows.

(P1) Uncorrelated prior $\Psi =\text{diag}\left(\sigma_{1}^{2},\ldots ,\sigma_{p}^{2}\right)$. An inverse prior is stipulated for the shrinkage parameters $\sigma_{j}^{2}$, *i.e.*, $p\left(\sigma_{j}^{2}\right)\propto \frac{1}{\sigma_{j}^{2}}$, j=1,…,p. Hence, the posterior distribution is $\sigma_{j}^{2}|\text{else}\propto \chi^{-2}\left(1,m_{j}^{2}\right)$. This approach is similar to that proposed by Xu (2003), which represents a baseline model.
(P2) Correlated prior $\Psi =K\sigma^{2}$. A flat prior is used for the variance component, *i.e.*, $p\left(\sigma^{2}\right)\propto \chi^{-2}\left(-2,0\right)$, which is similar to that proposed by Wang *et al.* (1994). This parameter is distributed *a posteriori* as $\sigma^{{}^{2}}|\text{else}∼\chi^{-2}\left(p-2,m^{\prime }K^{-1}m\right)$.
(P3) Correlated and adaptive prior Ψ=ΓKΓ with $\Gamma =\text{diag}\left(\gamma_{1},\ldots ,\gamma_{p}\right)$. The prior of the regularization parameters is specified as $p\left(\gamma_{j}\right)\propto \chi_{1}^{-2}$, j=1,…,p. Therefore, the pattern of the posterior density $p\left(\gamma_{j}|\text{else}\right)$ is unknown, but it is possible to employ a hybrid strategy, where a Metropolis step replaces the Gibbs step if sampling a parameter is impossible (Tierney 1994). For each *γ*, a single Metropolis iteration is used. A log-normal density is applied to the *γ*s as the proposal density $q\left(\gamma_{j}^{*}|\gamma_{j}^{\left(t-1\right)}\right)$ to obtain a random walk chain. By single-site updating during each iteration *t*, the *γ*s are individually drawn from the proposal distribution:
  $$x∼N\left(\text{ln}\gamma_{j}^{\left(t-1\right)},\varepsilon \right),$$
  $$\gamma_{j}^{*}=\text{exp}\left(x\right).$$

The tuning parameter *ε* is set to one. (The choice of *ε* is discussed below.) Let $\tau^{*}$ denote the vector of $\tau_{k}=1/\gamma_{k}$, k=1,…,p, where the *j*th component is replaced by the current proposed value, and $\tau^{\left(t-1\right)}$ refers to the vector of the samples obtained from the last iteration. The ratio *R* is obtained as (see *Appendix*):

$$\begin{array}{c} R=\frac{p\left(\gamma_{j}^{*}|\gamma_{-j}^{\left(t-1\right)},m^{\left(t-1\right)},y\right)}{q\left(\gamma_{j}^{*}|\gamma_{j}^{\left(t-1\right)}\right)}\frac{q\left(\gamma_{j}^{\left(t-1\right)}|\gamma_{j}^{*}\right)}{p\left(\gamma_{j}^{\left(t-1\right)}|\gamma_{-j}^{\left(t-1\right)},m^{\left(t-1\right)},y\right)}\\ =\frac{\text{exp}\left(-\frac{1}{2}\tau^{*\prime }\tilde{K}\tau^{*}+\frac{3}{2}\text{ln}\;\tau_{j}^{*}-\frac{\tau_{j}^{*}}{2}\right)}{\text{exp}\left(-\frac{1}{2}\tau^{{\left(t-1\right)}^{\prime }}\tilde{K}\tau^{\left(t-1\right)}+\frac{3}{2}\text{ln}\tau_{j}^{\left(t-1\right)}-\frac{\tau_{j}^{\left(t-1\right)}}{2}\right)}, \end{array}$$

with $\tilde{K}=K^{-1}\#mm^{\prime }$ using the Hadamard product (#). The acceptance ratio is then determined as α=min(R,1). The proposed value is accepted, $\gamma_{j}^{\left(t\right)}=\gamma_{j}^{*}$, if a random sample from a uniform distribution is lower than *α*; otherwise, $\gamma_{j}^{\left(t\right)}=\gamma_{j}^{\left(t-1\right)}$. The idea is related to the weighting of variables: with values starting at one, the proposed values of *γ* move slowly away from the initial estimate toward zero if there is evidence of a null effect, or they increase for nonzero SNP effects. Finally, after j=1,…,p Metropolis steps, the vector $m^{\left(t\right)}$ is sampled from the conditional distribution (3).

(P4) Correlated and adaptive prior $\Psi^{-1}=L\Gamma L^{\prime }$ with $\Gamma =\text{diag}\left(\gamma_{1},\ldots ,\gamma_{p}\right)$ and $LL^{\prime }=K^{-1}$. The prior of the shrinkage parameters is assumed to be $p\left(\gamma_{j}\right)\propto \chi_{1}^{2}$ for j=1,…,p. The posterior density is then recognized as the kernel of a scaled $\chi^{2}$ distribution with v=2 and $S={\left({\tilde{m}}_{j}^{2}+1\right)}^{-1}$, where $\tilde{m}=L^{\prime }m$ (see *Appendix*).

If covariances between SNPs are not considered, *i.e.*, K=I, the prior P2 is similar to a ridge-regression-type of model, while P3 and P4 are similar to, *e.g.*, BayesA-type of model (Meuwissen *et al.* 2001). In P3 and P4, two different types of modified Cholesky decompositions of the scale matrix K are incorporated (Pourahmadi 2007). For P3, where the correlations between SNPs are known and stationary, the covariances between SNPs are affected by the *γ*s which are sampled during Markov chain Monte Carlo (MCMC) simulations. Shrinkage based on P4 is related to differences in the effects of adjacent markers because the *γ*s influence the entries of the lower triangular matrix L.

Furthermore, the overall mean and residual variance component are distributed *a posteriori* as

$$\mu |\text{else}∼N\left(\frac{1}{n}1^{\prime }\left(y-\mathrm{Xm}\right),\frac{\sigma_{e}^{2}}{n}\right),$$

$$\sigma_{e}^{2}|\text{else}∼\chi^{-2}\left(n-2,e^{\prime }e\right)\;\text{with}\;\text{residuals}\;e=y-1\mu -\mathrm{Xm}.$$

The columns of X are centered to favor mixing of the MCMC algorithm (Stranden and Christensen 2011).

### Criteria for evaluation

The accuracy of genetic value prediction was verified by cross-validation. As a measure of accuracy, the mean correlation was calculated between the estimated and simulated genetic values in test data sets, $\rho =\text{cor}\left(X\hat{m},\mathrm{Xm}\right)$. In addition, to compare the predictive ability of the chosen priors, the mean true genetic value (TGV) was calculated for individuals, which were selected based on their estimated genetic value (EGV) according to a given fraction of selection (*r*). A range of *r* values between 0.05 and 1 was considered for evaluation. It is expected that the different methods would yield different rankings for the EGV. In general, the maximum mean TGV is obtained when only a few individuals are selected as parents of future offspring, and it approaches zero if more individuals are selected. Thus, the performance of a method can be determined by comparing the relationship between the mean TGV and *r*.

The precision of the estimates of the residual and genetic variance component was determined as the mean squared error (MSE) based on repeated simulations:

$$\text{MSE}\left(\sigma_{e}^{2}\right)=\frac{1}{B}\sum_{l=1}^{B}{\left({\hat{\sigma }}_{e}^{2\left(l\right)}-\sigma_{e}^{2}\right)}^{2},$$

$$\text{MSE}\left(\sigma_{a}^{2}\right)=\frac{1}{B}\sum_{l=1}^{B}{\left({\hat{\sigma }}_{a}^{2\left(l\right)}-\sigma_{a}^{2}\right)}^{2},$$

where ${\hat{\sigma }}_{e}^{2\left(l\right)}$ and ${\hat{\sigma }}_{a}^{2\left(l\right)}$ are the estimated residual and genetic variance in the *l*th training block, respectively, l=1,…,B. Given the estimated marker effects, the genetic variance was estimated as ${\hat{\sigma }}_{a}^{2}=\hat{m}\prime K\hat{m}$. The true genetic variance was calculated based on simulated marker effects as $\sigma_{a}^{2}=m^{\prime }\mathrm{Km}$ (Bonk *et al.* 2016). This formula considers the contribution of LD to the genetic variance (Gianola *et al.* 2013). Furthermore, the precision of the estimated effects at selected key SNPs was verified by the SD of traced samples based on one MCMC run. The SNPs at the five simulated quantitative trait loci (QTL) with largest effects and, for each of the two largest QTL, two SNPs being in high or low LD with the QTL, and within a window of 20 SNPs to both sides were selected as key SNPs.

An appropriate measure of significance was required to evaluate the suitability of the suggested priors for understanding the genetic architecture, particularly relevant genomic regions. First, because the SNP effects were correlated, segment effects $s_{j}^{\left(l\right)}=\sum_{k=0}^{10}m_{j+k}^{\left(l\right)}$ were calculated for a sliding window with an arbitrary width, which covered 11 SNPs, in the *l*th sampling round. These effects resemble haplotype effects. Second, the posterior probability of being positive was obtained from the Gibbs samples:

$$h_{j}=\frac{1}{N}\sum_{l=1}^{N}I\left(s_{j}^{\left(l\right)}>0\right),$$

where I(⋅) denotes the indicator function, and *N* the number of Gibbs samples after the burn-in period. If $h_{j}>0.95$ (positive effect size), or $h_{j}<0.05$ (negative effect size), the segment was declared to have a nonzero effect on y. This quantity represents a measure of evidence, which is a Bayesian analog of the *P*-value, in a similar manner to that described by De Braganca Pereira and Stern (1999).

### Data

Two sets of data were explored. First, simulated data were used to evaluate the performance of the Bayesian approach depending on the different prior choices. Second, real genotype data were used to study the pattern of covariance between SNPs in a real half-sib family and for a particular chromosome. A real phenotype was not analyzed because the present study focused on the impact of the covariance matrix on the outcomes of genomic evaluations. Thus, challenges related to real observations (other nuisance effects or uncertainty about genetic effects on the selected chromosome) were eliminated completely. Hence, a phenotype was simulated based on the real genotypes to investigate the feasibility of the method in a general manner.

#### Simulated data:

The genomic data were simulated using a synthetic and simplified approach, but the structure obtained for the dependencies resembled a realistic setting. Further details are provided in Supplemental Material, File S1. In total, 500 marker genotypes for 10,000 progeny were simulated on a chromosome segment with a length of 12 cM, but only the loci at which the sire was heterozygous were considered in further analyses (p=259). The SNP alleles were recoded, so the sire haplotypes were AA/BB regardless of the allele frequencies. The coding of alleles only affected the sign of the covariance and not the estimated effect size.

To simulate phenotypic observations, the effects of either five or 50 QTL were drawn randomly from a gamma distribution with shape parameter α=0.420, and scale parameter β=2.619. The sign was sampled with equal likelihood. In the five-QTL scenario, the second largest QTL was placed adjacent to the largest QTL, with four SNPs between them to complicate its detection. A residual error was added, and the resulting phenotype was scaled to have a variance of one. Finally, the simulated residual variance component $\sigma_{e}^{2}$ was 0.500, and, considering the formula for additive genetic variance given above, the simulated additive genetic variance $\sigma_{a}^{2}$ was 0.487 in the five-QTL scenario, and 0.489 in the 50-QTL scenario. The QTL were taken from the SNP set, and they remained in the data.

The accuracy of the predicted genetic value, and the estimated variance components was evaluated using a *B*-fold cross-validation. For that, the complete data set (n=10,000) was split into successive blocks with a training set size of n=100 (small sample size, B=100 repetitions) or n=1000 (medium sample size, B=10 repetitions).

#### Semireal data:

The data set comprised a single half-sib family of Holstein-Friesian cows (n=106), which were initially genotyped with a 50K SNP chip, where the complete data set was described by Wittenburg *et al.* (2013). The sire was phased based on the daughter genotypes using the R package *hsphase* (Ferdosi *et al.* 2014). For convenience, only p=903 SNPs at which the sire was heterozygous were selected from BTA1. The SNP alleles were recoded corresponding to the sire haplotypes AA/BB. The paternal recombination rate and LD of maternal gametes were estimated by numerical maximization (NM) of the log-likelihood function using the R function *optim* (R Core Team 2014) (see File S1). It was found that 407 of the 903 eigenvalues of K were negative, so the bending algorithm proposed by Jorjani *et al.* (2003) was employed to obtain a positive definite approximation of the covariance matrix. A phenotype was simulated based on five QTL ($\sigma_{a}^{2}=0.521$, $\sigma_{e}^{2}=0.500$), as described above.

The data are provided as File S2 and File S3. The physical order of the SNPs on BTA1 followed the Btau4.2 annotation (File S4).

### MCMC computing

(Metropolis-within-) Gibbs sampling algorithms were implemented in Fortran 90 embedding LAPACK 3.5.0 (www.netlib.org/lapack) and module *random* 1.13 (jblevins.org/mirror/amiller). The program ran on a 2.93 GHz multi-user system. A single chain comprising 50,000 sampling rounds was executed, and 20,000 iterations were omitted as the burn-in. The R package *coda* was used for MCMC diagnostics. In particular, the effective sample size (ESS) and Heidelberger and Welch’s test, which tests the null hypothesis that the samples were drawn from a stationary distribution, were employed to determine the convergence of a Markov chain.

### Data availability

The authors state that all data necessary for confirming the conclusions presented in the article are given in the Supplemental Material (File S2, File S3, and File S4).

## Results

### Simulated data

**Figure 1:**
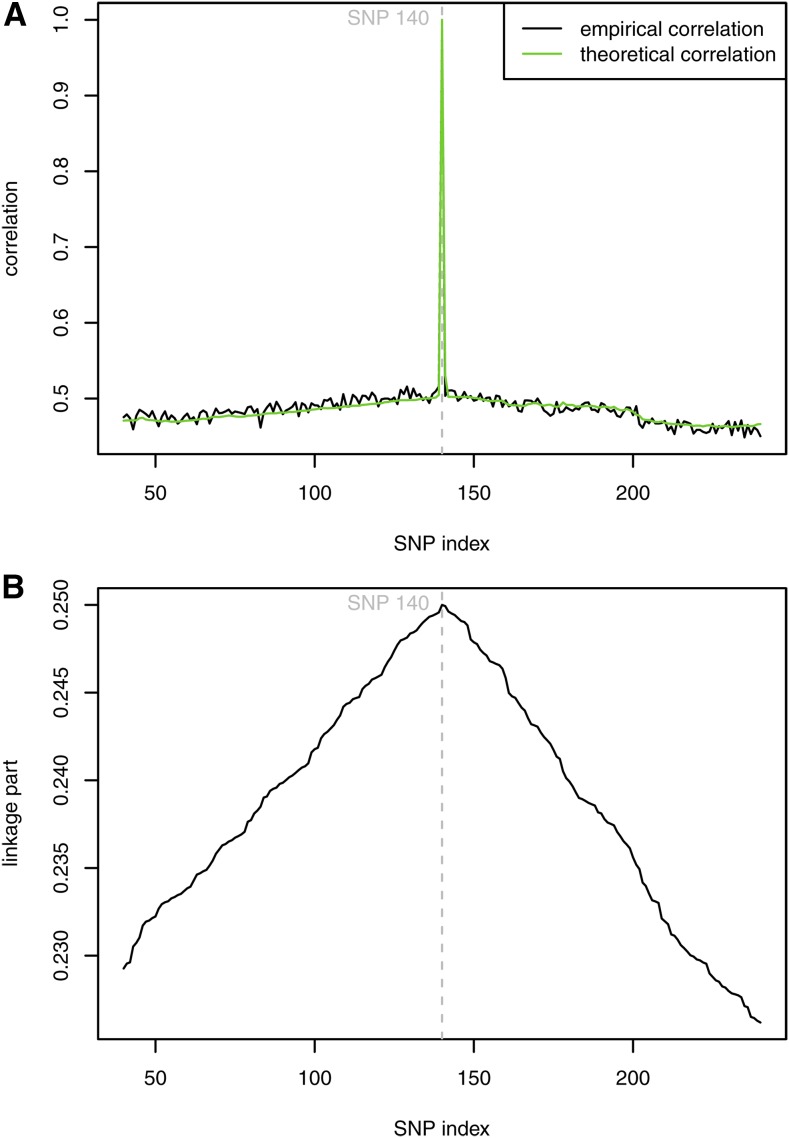
(A) Theoretical *vs.* empirical correlation for a randomly selected SNP based on the simulated genotypes. (B) Contribution of linkage (paternal part) to the covariance between SNPs.

The diagonal elements of the theoretical covariance matrix K ranged from 0.473 to 0.500, with a mean value of 0.492. The off-diagonal elements varied from 0.174 to 0.331 around a mean of 0.231. The covariance decreased gradually with increasing distance between the SNPs. After conversion into correlations, the off-diagonal entries ranged from 0.352 to 0.667, with a mean value of 0.470. As shown in Figure 1A, the theoretical and observed correlations were compared for a randomly selected SNP in a window of 200 SNPs, where the theoretical correlation decreased rapidly from the diagonal to off-diagonal elements, but this agreed with the values observed based on the genotypes. Figure 1B shows the paternal contribution (*i.e.*, linkage) to the covariance between SNPs, as discussed later.

MCMC diagnostics indicated the convergence of the MCMC algorithm for all of the proposed priors. ESS at key SNP effects was rather high, *i.e.*, > 10,309, and > 30,000 for a few exceptions; these values hardly differed between SNPs being the QTL or not. Heidelberger and Welch’s test was generally passed. The test failed only at the key SNP with the smallest simulated effect in the five-QTL scenario when the prior P4 was selected. Furthermore, for the hybrid strategy with prior P3, the acceptance rate *α* ranged from 0.54 to 0.79 (from 0.55 to 0.80) in the five-QTL (50-QTL) scenario.

**Figure 2:**
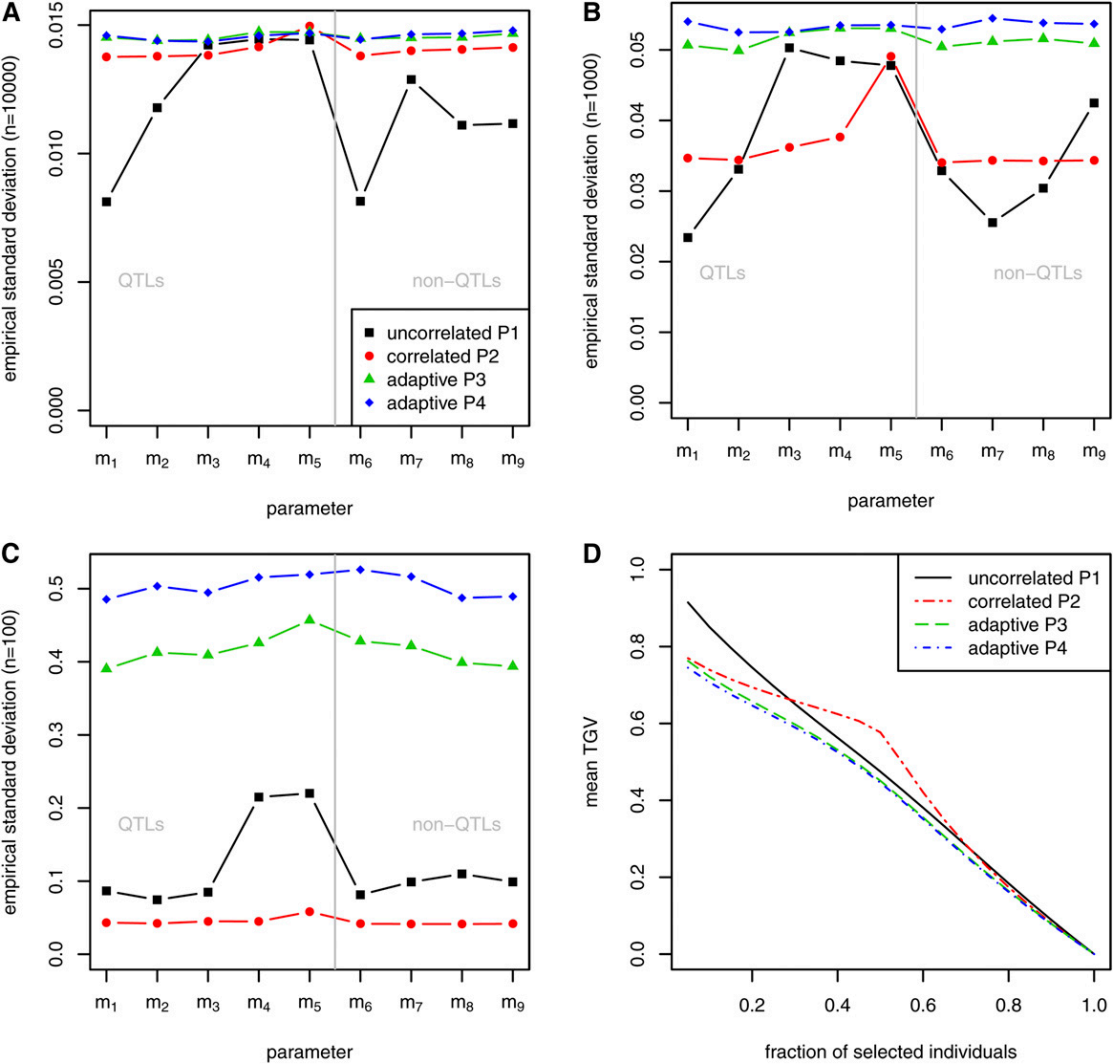
Simulation with five QTL. SD of estimated effects at key SNPs for different sample sizes based on one MCMC run: n=10,000 (A), n=1000 (B), n=100 (C). (D) Mean of TGV of individuals that were selected by their EGV based on 100-fold cross-validation (size of training set n=100).

For all of the selected priors, and with five and 50 simulated QTL, the estimated SNP effects agreed well with the simulated effects when the complete data set (n=10,000) was used. Most of the shrinkage effects were observed with the correlated prior P2. Many spurious effects were estimated with small effect sizes using all of the correlated prior choices. As expected, the empirical SD of key SNP effects was lowest when based on the complete data set, and it increased as the sample size decreased (*e.g.*, see Figure 2, A–C for the five-QTL scenario). In most cases, the SD of SNP effects at the QTL was lowest with the correlated prior P2, irrespective of the sample size. At non-QTL, smallest SD was obtained with P2 only for the small sample size. This was attributed to the very high shrinkage due to the prior P2.

**Figure 3:**
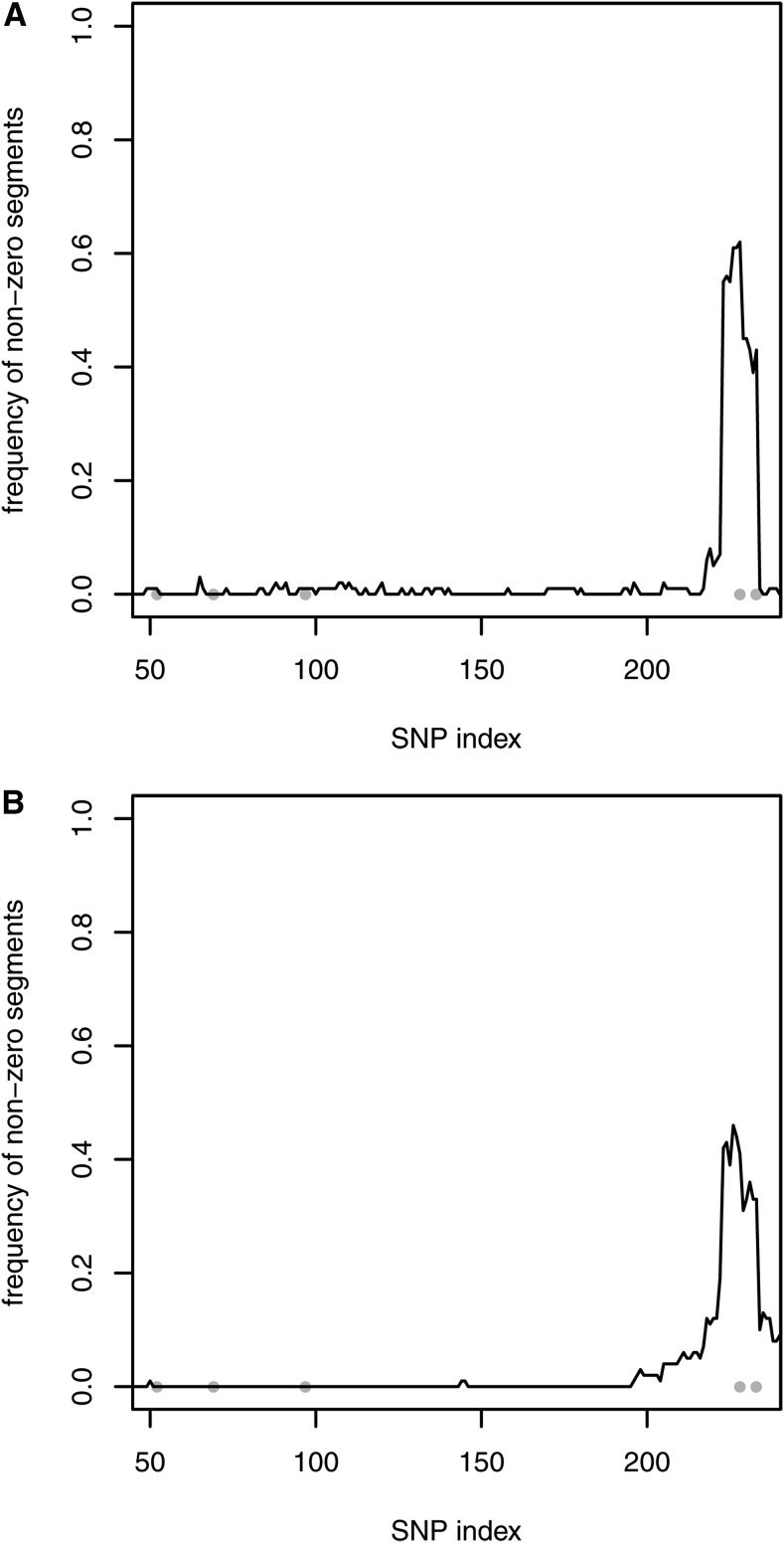
Simulation with five QTL, n=100, and 100 repetitions. Detection of nonzero segment effects using the uncorrelated prior P1 (A) and correlated prior P2 (B). Gray dots indicate the simulated QTL positions.

For the five-QTL scenario and based on one MCMC run, few significant chromosome segments were falsely detected when one of the correlated priors was selected. However, for small sample sizes, repeated simulations showed that using the uncorrelated prior P1 and correlated prior P2, only one significant segment at the end of the chromosome was identified correctly (Figure 3). The relevant region was smaller with the uncorrelated prior assumption. No significant segments were found with priors P3 and P4. For the 50-QTL scenario and using all priors, hardly any chromosome segments were detected due to the high level of uncertainty. For a medium sample size, the identification of significant segments was generally improved, and it even reached 100% for the largest QTL with all of the priors (results not shown).

The estimated variance components differed only slightly among the four methods for the complete data set, which were estimated to range from 0.475 to 0.497 for $\sigma_{a}^{2}$, and from 0.503 to 0.507 for $\sigma_{e}^{2}$ in the five-QTL scenario (Table 1). In the 50-QTL scenario, the estimates ranged from 0.499 to 0.522 for $\sigma_{a}^{2}$, and from 0.486 to 0.488 for $\sigma_{e}^{2}$ (Table 1). The MSE of the estimated variance components showed that the precision was greatest with the correlated prior P2 irrespective of the number of QTL when the sample size was small, as well as with a medium sample size and 50 QTL (see Table 2 and Table 3).

**Table 1:** Estimated variance components based on one MCMC run and n=10,000 observations

Prior	5 QTL		50 QTL	
	$\sigma_{e}^{2}$	$\sigma_{a}^{2}$	$\sigma_{e}^{2}$	$\sigma_{a}^{2}$
Uncorrelated P1	0.503	0.488	0.486	0.510
Correlated P2	0.507	0.475	0.488	0.499
Adaptive P3	0.506	0.497	0.488	0.522
Adaptive P4	0.506	0.497	0.488	0.522
Simulated	0.500	0.487	0.500	0.489

$\sigma_{e}^{2}$, residual variance; $\sigma_{a}^{2}$, additive genetic variance.

**Table 2:** Mean squared error (MSE) of the estimated variance components

Prior	5 QTL			50 QTL		
	MSE $\sigma_{e}^{2}$	MSE $\sigma_{a}^{2}$	*ρ*	MSE $\sigma_{e}^{2}$	MSE $\sigma_{a}^{2}$	*ρ*
Uncorrelated P1	0.145	0.074	0.760	0.157	0.080	0.747
Correlated P2	0.015	0.021	0.839	0.007	0.013	0.909
Adaptive P3	0.055	0.065	0.692	0.057	0.100	0.739
Adaptive P4	0.014	0.072	0.682	0.014	0.107	0.730

Correlation (*ρ*) between the predicted and simulated genetic values; 100-fold cross-validation (size of training set n=100). $\sigma_{e}^{2}$, residual variance; $\sigma_{a}^{2}$, additive genetic variance.

**Table 3:** MSE of the estimated variance components

Prior	5 QTL			50 QTL		
	MSE $\sigma_{e}^{2}$	MSE $\sigma_{a}^{2}$	*ρ*	MSE $\sigma_{e}^{2}$	MSE $\sigma_{a}^{2}$	*ρ*
Uncorrelated P1	0.00092	0.00116	0.974	0.00133	0.00358	0.964
Correlated P2	0.00090	0.00575	0.916	0.00042	0.00277	0.943
Adaptive P3	0.00056	0.01849	0.880	0.00069	0.02758	0.889
Adaptive P4	0.00060	0.02581	0.862	0.00047	0.03698	0.874

Correlation (*ρ*) between the predicted and simulated genetic values; 10-fold cross-validation (size of training set n=1000). $\sigma_{e}^{2}$, residual variance; $\sigma_{a}^{2}$, additive genetic variance.

The accuracy of the genetic value predictions was highest for the correlated prior P2 only with n=100; otherwise, the highest accuracy was obtained using the uncorrelated prior P1. The decrease in the mean TGV according to the fraction (*r*) of selected candidates gave mixed results (*e.g.*, see Figure 2D), where no method performed especially well with five QTL. The mean TGV was highest with the correlated prior P2 only when *r* was between about 30% and 70% in the five-QTL scenario, but this prior was generally better with 50 simulated QTL. The adaptive priors P3 and P4 generally performed worse in terms of all the evaluation criteria.

Additional figures showing the results are provided in File S1. Moreover, as part of this material, a simple simulation study was conducted to determine the shape of the theoretical covariance according to the recombination rate between a pair of SNPs. The average theoretical covariance agreed well with the empirical covariance obtained based on the progeny genotypes.

### Semireal data

**Figure 4:**
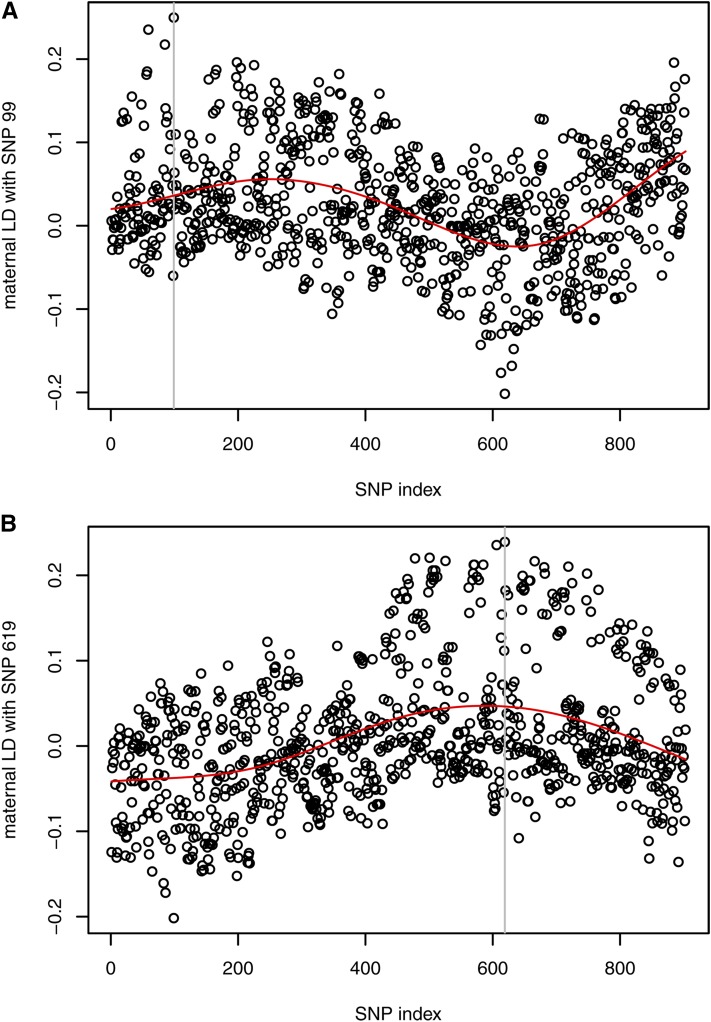
Estimated maternal LD with two selected SNPs on BTA1 for the real genotypes. The vertical line refers to the SNP with which pairwise LD was calculated. Smoothing via B-splines visualizes the trend of the data (red curves). (A) LD pattern shows a potential error in the marker map. (B) Maximum LD was observed around the reference SNP indicating correct positioning.

There was a good agreement between the theoretical and empirical correlations between SNP genotypes. The pattern of the correlations is shown in File S1. A gradual decrease in the entries was found with increasing distance between the SNPs. The off-diagonal values ranged from −0.411 to 1.000, with a mean value of 0.219. The low minimum value was due to an extreme estimate of the LD, *i.e.*, D=−0.202 between SNP 99 and 619, where the corresponding covariance was −0.193. If the distance between SNPs is rather large, then an extreme covariance indicates a potential error in the marker map. This hypothesis is supported by the values in the 99th row of D, which is the matrix of the pairwise LD values. It was expected that the maximum at around position 99 would decrease more or less smoothly on both sides, but this was not the case for this SNP, unlike SNP 619 (Figure 4).

Using MCMC computing, ESS at key SNP effects varied between 324 and 39,910, and was lowest with prior P2. However, ESS differed slightly between SNPs at the QTL and non-QTL. Heidelberger and Welch’s test was passed by all of the priors, which indicated that the Markov chain converged to a stationary distribution. Furthermore, the acceptance rate *α* ranged from 0.13 to 0.25 with prior P3.

The estimated SNP effects were far from their simulated effect sizes, which was due mainly to the small sample size causing a high amount of uncertainty. Using the correlated prior P2, which was the approach with the highest degree of shrinkage, a significant segment at the end of the chromosome was identified correctly. The variance components were estimated as $\sigma_{a}^{2}=0.419$ and $\sigma_{e}^{2}=0.505$. The other prior choices yielded variance component estimates > 2 × these values.

## Discussion

Our theoretical investigations have shown that the covariance between SNPs depends on the genetic distance and the maternal LD between them. In this study, the covariance matrix derived for a single half-sib family was employed in genomic evaluations of a simulated phenotype with heritability of about 50%. The results depended greatly on the sample size, but the inclusion of the covariance matrix was clearly beneficial for small sample sizes (n=100) in terms of both the MSE of the additive and residual variance components as well as the accuracy of the genetic value predictions, although there were no advantages with larger sample sizes (n=1000). These variable outcomes may have several explanations, which are related to the nature of covariance in the selected population design and the different prior assumptions.

### Covariance matrix

Previous studies have suggested that some covariance or correlation structure for marker effects should be included in regression models to estimate their effects. Candidates selected from covariance structures have proved useful in other fields, such as time series analysis, *i.e.*, equally spaced autoregressive, Toeplitz and Gaussian decay (Gianola *et al.* 2003), and first-order antedependence (Yang and Tempelman 2012). All of these methods assume some decay of the correlations with increasing distance between the markers, thereby considering the fact that recombination becomes more frequent when markers are more distant. However, in contrast to these previous methods, our derivation makes explicit use of well-established genetic arguments, and it is based on the recombination frequencies, Haldane’s underlying mapping function (Bonk *et al.* 2016), and the population-wide pairwise LD. Basically, the covariance between SNPs is determined as the sum of a paternal and maternal part. Assuming many half-sibs, the paternal part is considered for coinheritance (linkage), whereas the maternal part models the population-wide LD. The sign and size of the correlations are known *a priori* (with the exception of the second adaptive method, P4) in contrast to the antedependence model proposed by Yang and Tempelman (2012). In the latter model, the sign and size of the regression for a marker effect on that of its predecessor are determined during each iteration by sampling from a normal distribution. Both adaptive versions allow for some variation in the covariances because each sampled gamma parameter affects both the diagonal and off-diagonal elements of the inverse covariance matrix. In non-QTL regions, small *γ*s are expected, whereas large *γ*-values are related to QTL regions. This was also observed in our data analyses (see File S1). Therefore, such a covariance structure may be classified as nonstationary in a similar manner to the antedependence model because the covariances depend on the distances between markers and on the marker positions, *i.e.*, their proximity to QTL.

Unlike haplotype-based approaches (*e.g.*, Calus *et al.* 2008; Cuyabano *et al.* 2014), which require that all of the individuals are phased, only the sire’s haplotypes need to be known when using the proposed method. In the present study, we focused on a half-sib family structure, but other population structures are also relevant for livestock, *e.g.*, full-sib families often occur in chickens. Gametes of full-sibs are derived from a single sire and dam, and therefore the covariance between SNPs is restricted to the linkage part. (Paternal half-sibs represent a special case of a population with full-sib family structure, and the maternal gametes can be seen as random samples of a population with corresponding LD.) Then, the haplotypes of both parents should be considered when setting up the covariance matrix, as shown by Bonk *et al.* (2016). A typical livestock population comprises a mixture of families, so population-specific effects may be required instead of family-specific effects. The extension of the covariance matrix to a multiple-family study will be investigated in future research. The population covariance matrix could be set up as a weighted average of family-specific covariance terms, where the weight depends on the family size relative to the total sample size. Family sizes are often much smaller than 100, especially for full-sibs, so including the covariance between SNPs in genomic evaluations is expected to be beneficial, but this should be confirmed in a subsequent study.

All of the parameters required for the covariance matrix K were available for the simulated data. However, the recombination rate and LD of the maternal gametes had to be estimated from the progeny genotypes for the real data. In this study, estimates were obtained by NM of the log-likelihood function. Alternatively, *θ* and *D* could be estimated by the expectation-maximization (EM) algorithm (Gomez-Raya *et al.* 2013). However, this iterative approach is more time consuming than the iterations required for NM. There was good agreement between the theoretical and empirical covariances obtained from the progeny genotypes, but the covariance matrix was indefinite for the real data genotypes (and positive definite for the simulated data), and thus it was forced to be positive definite by bending. This was probably caused by errors in the estimation of the required parameters, which could be estimated more precisely using larger families.

When the alleles of the sire haplotypes were coded according to the observed minor allele frequency in the sample, both of the sire diplotypes AA/BB and AB/BA appeared in the double heterozygous case. The covariance matrix had then a block structure, which represented the nonrecombinant genome segments.

### Prior assumptions

**Figure 5:**
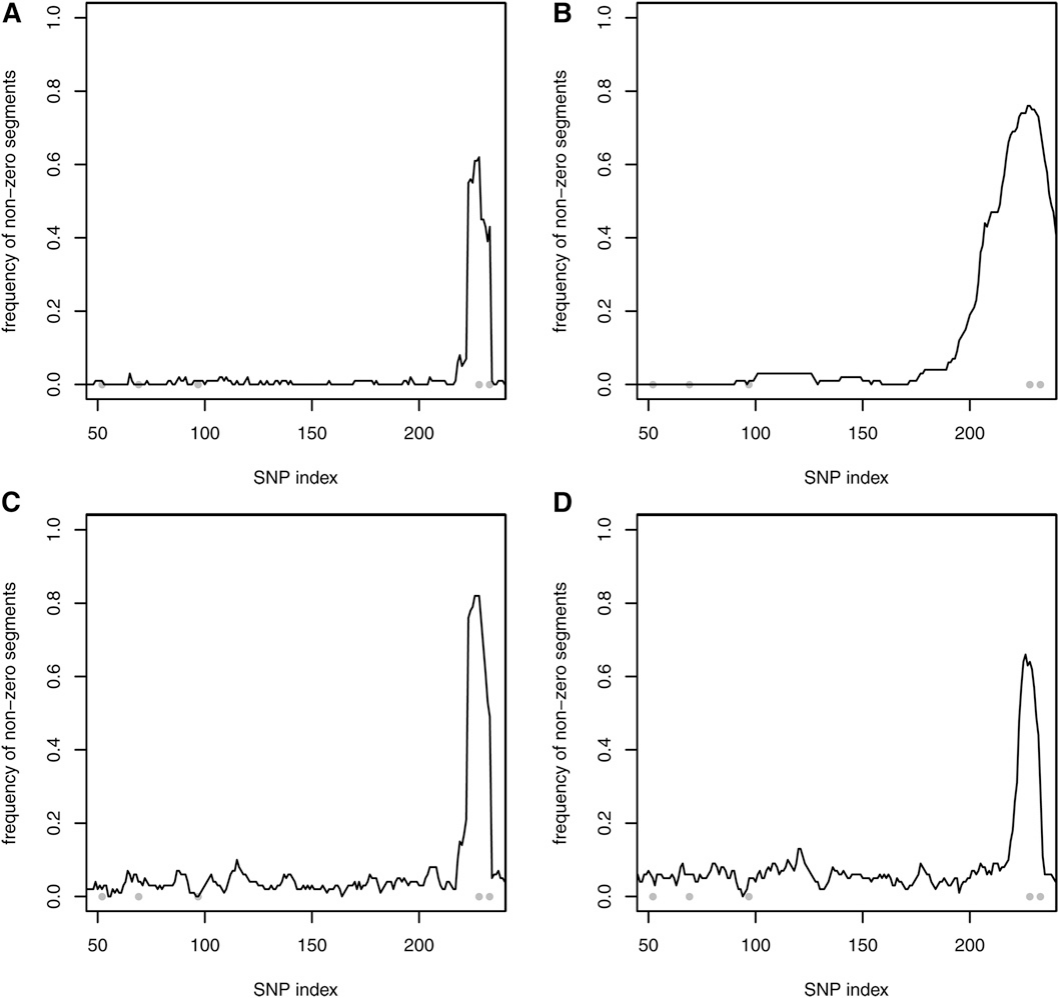
Simulation with five QTL, n=100, and 100 repetitions. Detection of nonzero segment effects using the **sparse** inverse covariance matrix and uncorrelated prior P1 (A), correlated prior P2 (B), adaptive prior P3 (C), and adaptive prior P4 (D). Gray dots indicate the simulated QTL positions.

In this study, each of the correlated priors incorporated a dense covariance matrix, and its inverse is also dense. According to Lang *et al.* (2002), locally adaptive random-walk priors penalize the difference between successive effects, thereby allowing local regularization to avoid oversmoothing. The inverse $K^{-1}$, which is also called the precision matrix, could be adjusted to have a banded structure to allow better local adaptivity in genomic evaluations. As an option, sparseness can be achieved by eliminating the “noise”. In the present study, the paternal contribution (*i.e.*, linkage) to the covariance matrix was more important than the maternal part (*i.e.*, population LD), where the interquartile range of *D* was zero in the simulations (mean =0) and 0.064 in the real genotypes (mean =0.016). Thus, an approximation of K retained only the paternal part ${\left\{\frac{1}{4}\left(1-2\theta_{j,k}\right)\right\}}_{j,k=1}^{p}$. Figure 1B shows the pattern for a randomly selected SNP. This simple structure can be inverted theoretically by exploiting, for instance, $1-2\theta_{1,3}=\left(1-2\theta_{1,2}\right)\left(1-2\theta_{2,3}\right)$, based on Haldane’s mapping function. The dependence throughout the genome represents an autoregressive process, so the inverse covariance matrix depends only on the predecessor and successor, thereby yielding a three-band matrix that fully considers the unequal distances between SNPs. This type of sparse structure has also been derived and implemented using a Gibbs sampling strategy for a backcrossed population by Reichelt *et al.* (2015). Using this structure, a more local impact of the regularization parameters is obtained using the adaptive priors P3 and P4, and the error propagation caused by numerical imprecisions along the chromosome appears to be reduced. The influence of this structure on the performance of the correlated prior selections is shown in Table 4 and Table 5, and in File S1. In particular, the MSE was smallest using the adaptive priors P3 and P4. Furthermore, the identification of significant chromosome segments was improved, and slightly superior to the uncorrelated prior P1 (*e.g.*, see Figure 5 for the five-QTL scenario). During the analysis of real data, this approximation has an additional positive impact because only the recombination rate needs to be estimated. Unlike classical linkage analysis or linkage/LD analysis (*e.g.*, Meuwissen *et al.* 2002), which is applied to a marker bracket instead of all markers simultaneously, this restriction to the linkage part, works well, even though the parental origin of the SNP alleles has not been identified.

**Table 4:** MSE of the estimated variance components when the inverse covariance matrix was sparse

Prior	5 QTL			50 QTL		
	MSE $\sigma_{e}^{2}$	MSE $\sigma_{a}^{2}$	*ρ*	MSE $\sigma_{e}^{2}$	MSE $\sigma_{a}^{2}$	*ρ*
Uncorrelated P1	0.145	0.074	0.760	0.157	0.080	0.747
Correlated P2	0.036	0.022	0.831	0.014	0.013	0.904
Adaptive P3	0.087	0.053	0.779	0.083	0.065	0.772
Adaptive P4	0.010	0.014	0.745	0.011	0.029	0.795

Correlation (*ρ*) between the predicted and simulated genetic values; 100-fold cross-validation (size of training set n=100). For comparison, results with P1 were taken from Table 2.$\;\sigma_{e}^{2}$, residual variance; $\sigma_{a}^{2}$, additive genetic variance.

**Table 5:** MSE of the estimated variance components when the inverse covariance matrix was sparse

Prior	5 QTL			50 QTL		
	MSE $\sigma_{e}^{2}$	MSE $\sigma_{a}^{2}$	*ρ*	MSE $\sigma_{e}^{2}$	MSE $\sigma_{a}^{2}$	*ρ*
Uncorrelated P1	0.00092	0.00116	0.974	0.00133	0.00358	0.964
Correlated P2	0.01499	0.01413	0.858	0.00563	0.00465	0.911
Adaptive P3	0.00084	0.00110	0.969	0.00116	0.00390	0.960
Adaptive P4	0.00063	0.00070	0.965	0.00045	0.00266	0.951

Correlation (*ρ*) between the predicted and simulated genetic values; 10-fold cross-validation (size of training set n=1000). For comparison, results with P1 were taken from Table 3. $\sigma_{e}^{2}$, residual variance; $\sigma_{a}^{2}$, additive genetic variance.

A second option for approximation uses the modified Cholesky decomposition of the inverse matrix, $\Psi^{{}^{-1}}=L\Gamma L^{\prime }$, which is suitable for P4. Wu and Pourahmadi (2003) derived a smoothing algorithm for L along its subdiagonals for longitudinal data and p<n. Future studies should investigate whether this banded structure can also be derived for p>n. The sparse structure of the inverse matrix is also a consequence of the antedependence specification proposed by Yang and Tempelman (2012). Exploiting a sparse structure in the MCMC algorithm will also improve the computational speed (*e.g.*, for n=100, the current implementation required on average 15 min based on prior P1, and 48 min based on prior P4.)

The prior covariance matrix used in P3 is similar to that described by George and McCulloch (1993) in terms of the dynamic regularization parameters (*γ*s), although it is implemented as a variable selection approach. In the present study, variable selection was not executed because it was assumed that each marker contributes to the genetic variation, at least indirectly through LD. However, this strategy led to poor mixing in the MCMC algorithm compared with the adaptive prior P4 using semireal data (results not shown). The trace plots contained repetitions of effect samples with low variability, followed by samples with very high variability, which was not observed to the same extent for the simulated data with small sample sizes. The correlated prior P2 was the most robust prior choice for both the simulations and semireal data because it could detect significant chromosome segments and estimate the variance components with the smallest MSE. To facilitate realistic genome-wide evaluations, we recommend specifying chromosome-wise priors, *i.e.*, $m_{c}|\Psi_{c}∼N\left(0,\Psi_{c}\right)$ and $\Psi_{c}=K_{c}\sigma_{c}^{2}$ for $c=1,\ldots ,n_{\text{chr}}$. Genome-wide shrinkage, which employs a single regularization parameter $\sigma^{2}$, may be too strict if only a few QTL are present.

Bayesian approaches require the specification of prior distributions but the use of hyper-parameters was minimized in the present study. The sensitivity to the selected priors has been evaluated and discussed previously, *e.g.*, by Knürr *et al.* (2013) and Gianola (2013). In the adaptive prior P3, the tuning variance was set to ε=1.0, which made this approach sensitive, and it compromised the repeatability of the experiments. For example, for different choices of ε∈{0.01,0.1,1.0}, a varying acceptance rate *α* was obtained, where *α* was higher when *ε* was smaller. However, the impact on a single-effect estimate was negligible for both the simulated and semireal data, whereas the estimated variance components $\sigma_{e}^{2}$ and $\sigma_{a}^{2}$ differed in terms of the second decimal place (results not shown). With ε=1.0, the range of *α* roughly agreed with the rule of thumb given by Besag *et al.* (1995) who showed that an acceptance rate between about 30% and 70% was often satisfactory. Alternatively, the tuning variance may be adjusted during the burn-in phase to obtain an intermediate *α* (25–50%) in a similar manner to Yang *et al.* (2015).

### Sample size

The sample size had a major effect on the outcome. For small sample sizes (n=100), the effect estimates were poor, and a high EGV was obtained even for those individuals with low TGV. Thus, the curves of the mean TGVs (*e.g.*, in Figure 2D based on five simulated QTL) started below 0.900 at a selection rate r=5%, although the 95% quantile for the TGV was 1.106. The shape of the curve based on the correlated prior choice P2 was unusual, where it decreased very slightly until r=50%, and it then declined rapidly, which may be explained by the following two reasons. First, the SNP effects were greatly reduced toward zero due to the major effect of the shrinkage parameter $\sigma^{2}$. Second, the sire was heterozygous at all of the loci considered (with A alleles on one strand and B alleles on the other), so there were only few recombinant offspring, and the distribution of EGV was bimodal (see File S1). The variation around the two modes was due to variation in the maternally inherited gametes. Thus, the individuals selected with the highest EGV comprised a mixture of high/medium/low-TGV individuals, and the curve obtained for the mean TGVs decayed slowly. The curve approached the population mean rapidly above r=50 %. This outcome was also observed, but to a much greater extent, in simulations with 50 QTL as well as when combined with the sparse inverse covariance matrix (File S1). This pattern was not detected with a medium sample size (n=1000), probably because of the improved estimation of the parameters, and the occurrence of more recombinant offspring. This phenomenon is unlikely to occur if more than one chromosome is investigated, thereby causing greater variation in the paternal gametes.

### Conclusion

In this study, the dependence between SNP genotypes was derived theoretically from the genetic parameters for a half-sib family. Integrating this information into genomic evaluations improved the estimates of the variance components and the genetic value predictions when the sample size was small (n=100). Thus, in small populations, for which the parameter estimates are typically affected by high uncertainty when the number of predictors is large, additional information about the population structure can increase the precision, thereby following the general Bayesian principle. The proposed correlated prior choices could potentially obtain better overall performance if a locally adaptive approximation of dependence is employed.

## Supplementary Material

Supplemental Material

## Appendix

### Prior P3

The target distribution $p\left(\gamma_{j}|\text{else}\right)$ is obtained from

$$\begin{array}{c} p\left(\gamma_{j}|\gamma_{-j},m,y\right)\propto p\left(m|\gamma \right)p\left(\gamma \right)\\ \propto p\left(m|\gamma \right)p\left(\gamma_{j}\right)\\ \propto {\left|\Psi \right|}^{-1/2}\text{exp}\left(-\frac{1}{2}m^{\prime }\Psi^{-1}m\right)\gamma_{j}^{-3/2}\text{exp}\left(-\frac{1}{2\gamma_{j}}\right). \end{array}$$

For convenience, it is $\tau_{j}=\frac{1}{\gamma_{j}}$ and $\tau ={\left(\tau_{1},\ldots ,\tau_{p}\right)}^{\prime }$. Hence,

$$\begin{array}{c} m^{\prime }\Psi^{-1}m=m^{\prime }\Gamma^{-1}K^{-1}\Gamma^{-1}m\\ =\text{tr}\left(K^{-1}\Gamma^{-1}mm^{\prime }\Gamma^{-1}\right)\\ =\sum_{j=1}^{p}\sum_{k=1}^{p}K_{j,k}^{-1}\frac{m_{j}}{\gamma_{j}}\frac{m_{k}}{\gamma_{k}}\\ =\sum_{j=1}^{p}\sum_{k=1}^{p}{\tilde{K}}_{j,k}\tau_{j}\tau_{k}\\ =\tau^{\prime }\tilde{K}\tau \end{array}$$

with $\tilde{K}=K^{-1}\#mm^{\prime }$ employing the Hadamard product (#). Thus,

$$p\left(\gamma_{j}|\gamma_{-j},m,y\right)\propto \tau_{j}\exp\left(-\frac{1}{2}\tau^{\prime }\tilde{K}\tau +\frac{3}{2}\text{ln}\;\tau_{j}-\frac{\tau_{j}}{2}\right).$$

The proposal density is lognormal, *i.e.*,

$$q\left(\gamma_{j}^{*}|\gamma_{j}^{\left(t-1\right)}\right)={\left(2\pi \varepsilon \right)}^{-1/2}\text{exp}\left(-\frac{1}{2\varepsilon }{\left(\text{ln}\;\gamma_{j}^{*}-\text{ln}\;\gamma_{j}^{\left(t-1\right)}\right)}^{2}\right)\frac{1}{\gamma_{j}^{*}}.$$

Except the last ratio, this function is symmetric in $\gamma_{j}^{*}$ and $\gamma_{j}^{\left(t-1\right)}$. Let $\tau^{*}$ denote the vector of *τ*s, where the *j*th component is replaced by the current proposed value, and $\tau^{\left(t-1\right)}$ refers to the vector of samples from the last iteration. Finally, the ratio *R* is

$$\begin{array}{c} R=\frac{p\left(\gamma_{j}^{*}|\gamma_{-j}^{\left(t-1\right)},m^{\left(t-1\right)},y\right)}{q\left(\gamma_{j}^{*}|\gamma_{j}^{\left(t-1\right)}\right)}\frac{q\left(\gamma_{j}^{\left(t-1\right)}|\gamma_{j}^{*}\right)}{p\left(\gamma_{j}^{\left(t-1\right)}|\gamma_{-j}^{\left(t-1\right)},m^{\left(t-1\right)},y\right)}\\ =\frac{\text{exp}\left(-\frac{1}{2}\tau^{*\prime }\tilde{K}\tau^{*}+\frac{3}{2}\text{ln}\;\tau_{j}^{*}-\frac{\tau_{j}^{*}}{2}\right)}{\text{exp}\left(-\frac{1}{2}\tau^{{\left(t-1\right)}^{\prime }}\tilde{K}\tau^{\left(t-1\right)}+\frac{3}{2}\text{ln}\;\tau_{j}^{\left(t-1\right)}-\frac{\tau_{j}^{\left(t-1\right)}}{2}\right)}\;. \end{array}$$

### Prior P4

The posterior density of regularization parameters is derived as

$$\begin{array}{c} p\left(\gamma_{1},\ldots ,\gamma_{p}|\text{else}\right)\propto p\left(m|\gamma \right)p\left(\gamma \right)\\ \propto {\left|\Psi \right|}^{1/2}\text{exp}\left(-\frac{1}{2}m^{\prime }\Psi^{-1}m\right)\prod_{j=1}^{p}\gamma_{j}^{-1/2}\text{exp}\left(-\frac{\gamma_{j}}{2}\right)\\ \propto \text{exp}\left(-\frac{1}{2}\text{tr}\left(\Gamma L^{\prime }mm^{\prime }L\right)\right)\prod_{j=1}^{p}\text{exp}\left(-\frac{\gamma_{j}}{2}\right). \end{array}$$

Set $\tilde{m}=L^{\prime }m$, then

$$\begin{array}{c} p\left(\gamma_{1},\ldots ,\gamma_{p}|\text{else}\right)\propto \text{exp}\left(-\frac{1}{2}\sum_{j=1}^{p}\gamma_{j}{\tilde{m}}_{j}^{2}\right)\prod_{j=1}^{p}\text{exp}\left(-\frac{\gamma_{j}}{2}\right)\\ =\prod_{j=1}^{p}\text{exp}\left(-\frac{1}{2}\gamma_{j}\left({\tilde{m}}_{j}^{2}+1\right)\right). \end{array}$$

Thus, the *γ*s are conditionally independent, each distributed as scaled $\chi^{2}$.
