# Supplementary material for: Covariance Between Genotypic Effects and its Use for Genomic Inference in Half-Sib Families
Source: G3 (Bethesda). 2016 Jul 7;6(9):2761–72. doi: 10.1534/g3.116.032409 (PMC5015933; doi:10.1534/g3.116.032409)
Supplement: Supplemental Material [file supp_6_9_2761__index.html]

Covariance Between Genotypic Effects and its Use for Genomic Inference in Half-Sib Families — Supplemental Material 

# Covariance Between Genotypic Effects and its Use for Genomic Inference in Half-Sib Families

## Supplemental Material for Wittenburg *et al.*, 2016

**Files in this Data Supplement:**

- File S1 - Supplemental material and additional figures. (.pdf, 1 MB)
- File S2 - Compressed file containing the simulated data (SNP genotypes, TGV, phenotype, and covariance matrix). (.zip, 1 MB)
- File S3 - Compressed file containing the semi-real data (SNP genotypes, TGV, phenotype, and covariance matrix). (.zip, 4 MB)
- File S4 - Physical order of SNPs on BTA1 according to the Btau4.2 annotation. (.txt, 92 KB)
