# Supplementary material for: Covariance Between Genotypic Effects and its Use for Genomic Inference in Half-Sib Families
Source: G3 (Bethesda). 2016 Jul 7;6(9):2761–72. doi: 10.1534/g3.116.032409 (PMC5015933; doi:10.1534/g3.116.032409)
Supplement: Supplemental Material [file supp_g3.116.032409_FileS1.pdf]

S Supplementary material

S.1 Data simulation

The (paternal) genetic distances and maternal haplotype frequencies were sampled directly to determine the true entries of $\boldsymbol{K}$. The procedure of simulating the marker genotypes can be divided into four steps. First, since non-equidistantly distributed marker positions are desired, distances between 500 markers were sampled from a generalized inverse normal distribution with parameters $h = 10^{-7}$ and $a = b = 1$. Afterwards, distances were scaled by 0.01, and 0.01 was added to obtain more realistic values which were interpreted as cM. Second, the marker haplotypes of the sire were simulated by sampling alleles from a Bernoulli distribution $B(p_j)$ with $p_j = 0.6$ for all loci $j = 1, \ldots, 500$. Third, paternal gametes were generated. The allele at the first locus was drawn by chance with the probability 0.5. The recombination rate $\theta_{j,j+1}$ between the current and the next locus was then calculated using the simulated marker map and Haldane's mapping function. Sampling from $B(\theta_{j,j+1})$ determined whether a recombination event happened or not. Fourth, the maternal gametes were simulated. The haplotype frequencies $p^{AA}_{j,j+1}$, $p^{AB}_{j,j+1}$, $p^{BA}_{j,j+1}$ and $p^{BB}_{j,j+1}$ between neighbouring loci j and $j+1$ were first sampled from a log-normal distribution with $\mu = 0.25$ and $\sigma = 0.001$ to ensure positivity. These values were normalized afterwards, such that they sum up to one for each locus j. Further conditions have to hold, e.g.,

$$
\begin{aligned}
p^{AA}_{j,j+1} + p^{BA}_{j,j+1} \;=\; p^{A}_{j+1} &\;=\; p^{AA}_{j+1,j+2} + p^{AB}_{j+1,j+2} \\
p^{A}_{1} &\;=\; p^{AA}_{1,2} + p^{AB}_{1,2} \,.
\end{aligned}
$$

Once all haplotype frequencies have been simulated, the allele at locus j on the maternal gamete was obtained by sampling from $B(p^{A}_{j})$. In total, 500 marker genotypes of 10 000 progeny were simulated but only the loci at which the sire was heterozygous were considered in further analyses ($p = 259$). The total length of the simulated chromosome

segment was 12 cM.

S.2 Shape of covariance between SNP genotype codes

A small simulation study was conducted to analyze the pattern of the covariance function between two SNPs. The sire of 1 000 half-sibs was assumed to be double heterozygous (haplotypes AA and BB). Assuming that the dam population descended after 20 generations from an F_2 population, the parameters of the dam population were: $p_1^A = 0.7$, $p_2^A = 0.6$ and $D_{1,2} = (1 - \theta_{1,2})^{20} D_0$ with $D_0 = (1 - 2\theta_{1,2})/4$. The two haplotypes of a dam were randomly drawn with the probabilities $(p_{1,2}^{AA}, p_{1,2}^{AB}, p_{1,2}^{BA}, p_{1,2}^{BB})$ with, for instance, $p_{1,2}^{AA} = p_1^A p_2^A + D_{1,2}$. Then, haplotypes of a progeny were drawn by chance with respect to the recombination rate $\theta_{1,2} \in \{0\,(0.01)\,0.5\}$ which was assumed to be equal for the maternal and paternal haplotypes. Note that D refers here to the LD of the dam population and not the LD of maternal gametes as in the main paper. The simulation was repeated 100 times.

The shape of the covariance function between two loci depending on the recombination rate is shown in Figure Aa; the larger $\theta_{1,2}$ is, the less covariance is expected. This theoretical curve is confronted with the empirical covariance between the observed SNP genotypes in each simulation. The empirical covariance coincided well on average with the theoretical covariance. In a different scenario with at least one homozygous loci at the sire, the covariance function decreases faster (Figure Ab).

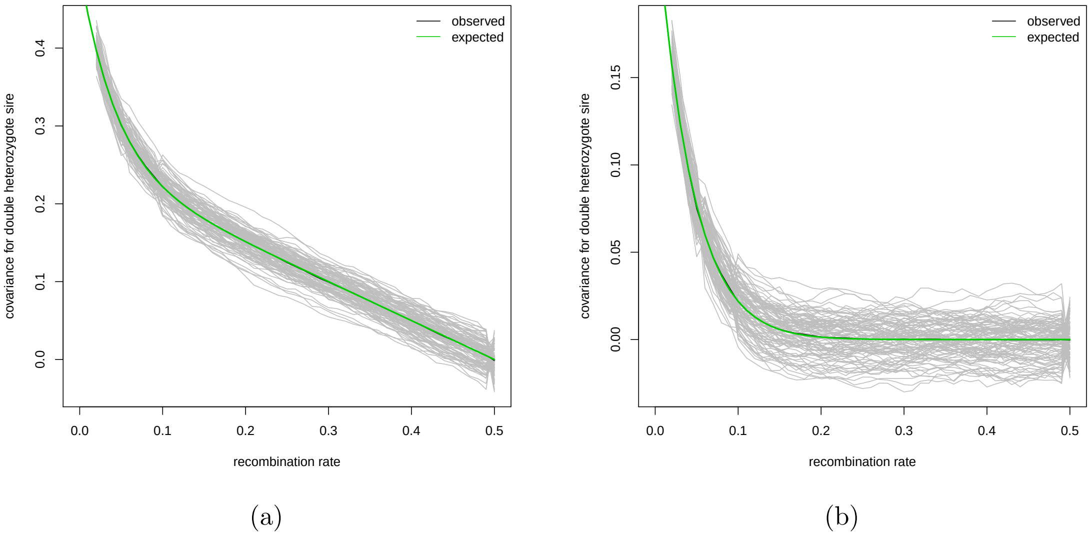

Figure A Empirical covariance between the genotype codes at two loci (one gray curve for each repeated simulation) vs. theoretical covariance between two loci depending on the recombination rate (green curve); (a) sire haplotypes AA and BB; (b) sire haplotypes AA and AB.

S.3 Estimation of paternal recombination rate and maternal linkage disequilibrium

For real data, where only the progeny genotypes are observable, the parameters of interest D and θ required for $\boldsymbol{K}$ can be estimated by numerical maximization of the log-likelihood function at a given SNP pair (j,k). A double heterozygous sire AA/BB is assumed, but the notes below can be adapted to other diplotypes. For convenience, the indices at D and θ are omitted. The maximization problem is

$$\max_{L_1<D<L_2, 0<\theta<1} \ell(D,\theta|\boldsymbol{X}_{\cdot,j},\boldsymbol{X}_{\cdot,k})$$

with $\boldsymbol{X}_{\cdot,j}$ and $\boldsymbol{X}_{\cdot,k}$ being the jth and kth column, respectively, of the genotype matrix $\boldsymbol{X}$, and the log-likelihood function is calculated by

$$\begin{aligned}
\ell(D,\theta|\boldsymbol{X}_{\cdot,j},\boldsymbol{X}_{\cdot,k}) &= \sum_{g\in\{1,0,-1\}}\sum_{h\in\{1,0,-1\}} n_{g,h}\log(p_{g,h})\,, \\
p_{g,h} &= \Pr(X_{i,j}=g \wedge X_{i,k}=h)\,, \\
n_{g,h} &= \frac{1}{n}|\{i : X_{i,j}=g \wedge X_{i,k}=h\}|\,.
\end{aligned}$$

As an example, for $g=h=0$, i.e., the offspring have a double heterozygous genotype, it is

$$p_{0,0} = \frac{1-\theta}{2}\left(p_{j,k}^{BB}+p_{j,k}^{AA}\right)+\frac{\theta}{2}\left(p_{j,k}^{AB}+p_{j,k}^{BA}\right)\,,$$

or for a double homozygous sire, $g=1$ and $h=-1$,

$$p_{1,-1} = \frac{\theta}{2}p_{j,k}^{AB}\,.$$

For the optimization problem with given bounds, the lower (L_1) and upper limit (L_2) of the maternal LD are derived from the haplotype probabilities:

$$
\begin{aligned}
L_1 &= \max\left\{-p_j^A p_k^A, -(1-p_j^A)(1-p_k^A)\right\} , \\
L_2 &= \min\left\{p_j^A(1-p_k^A), (1-p_j^A)p_k^A\right\} .
\end{aligned}
$$

Only the loci at which the sire is heterozygous are considered, the maternal allele frequencies are then estimated as $p_j^A = \frac{1}{n}\sum_{i=1}^{n} X_{i,j} - \frac{1}{2}$.

Finally, the R function *optim* with method "L-BFGS-B" (which is a box-constrained version of a quasi-Newton method developed by Broyden, Fletcher, Goldfarb and Shanno) was employed to solve the maximization problem.

S.4 Additional figures

The following figures show the results for the simulation study based on either five or 50 QTLs and varying sample sizes $n \in \{100, 1\,000, 10\,000\}$. The results based on the semi-real data set ($n = 106$, 5 QTLs) show a high level of uncertainty due to $p >> n$.

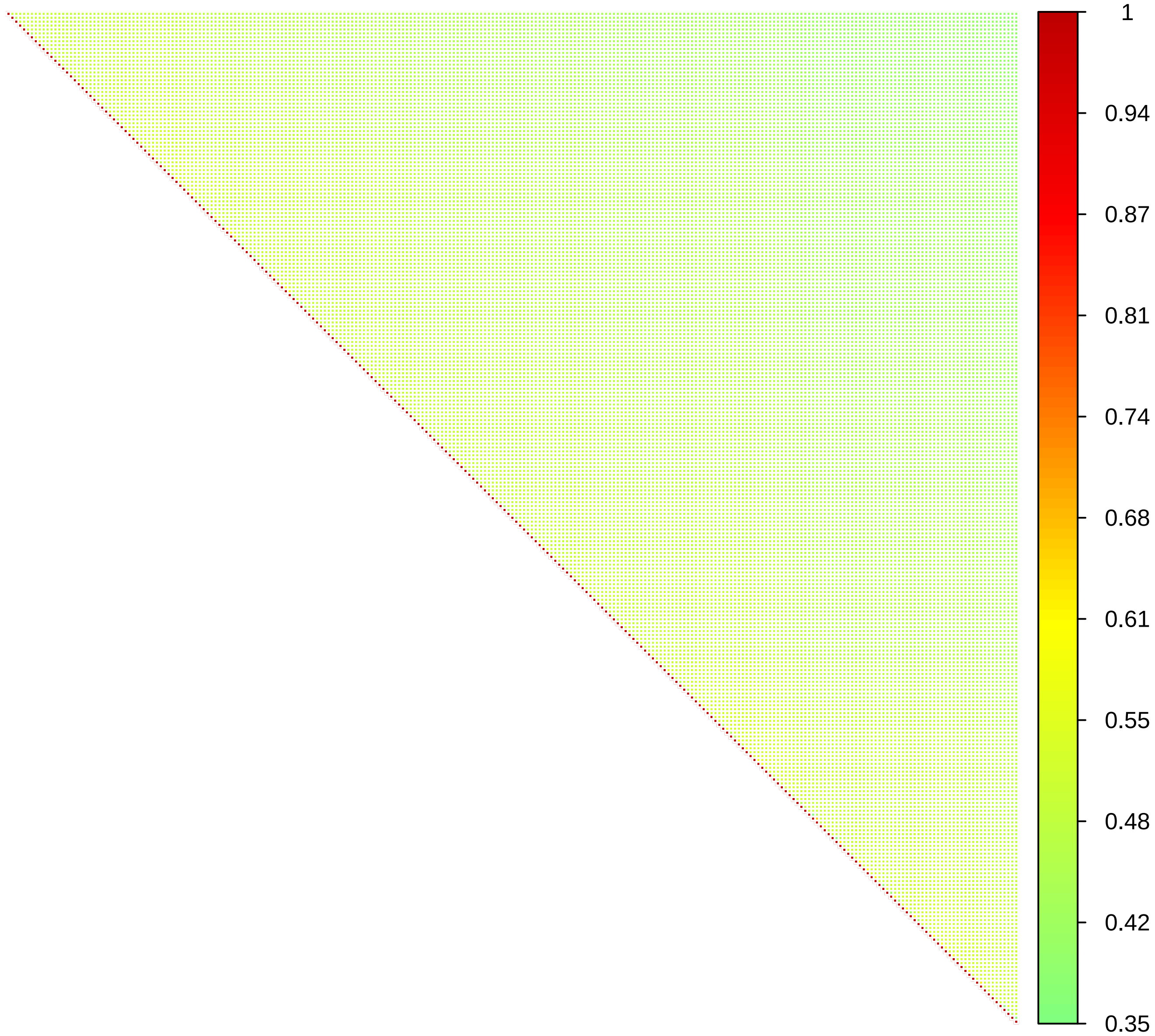

Figure B Theoretical correlation matrix of SNP 1–259 for the simulated genotypes.

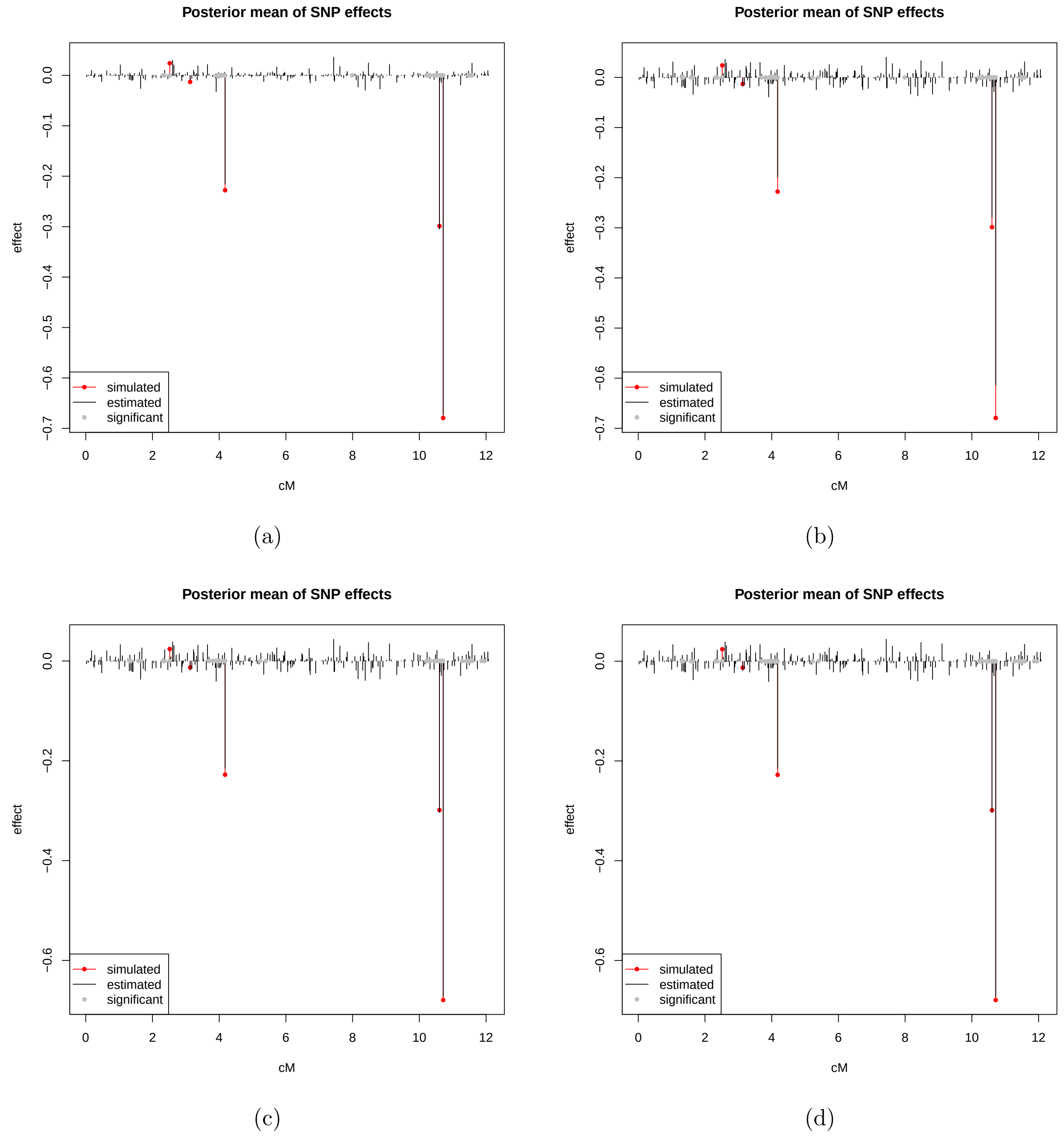

Figure C Simulation with five QTLs and $n = 10\,000$. Estimated SNP effects using the (a) uncorrelated prior P1, (b) correlated prior P2, (c) adaptive prior P3, (d) adaptive prior P4.

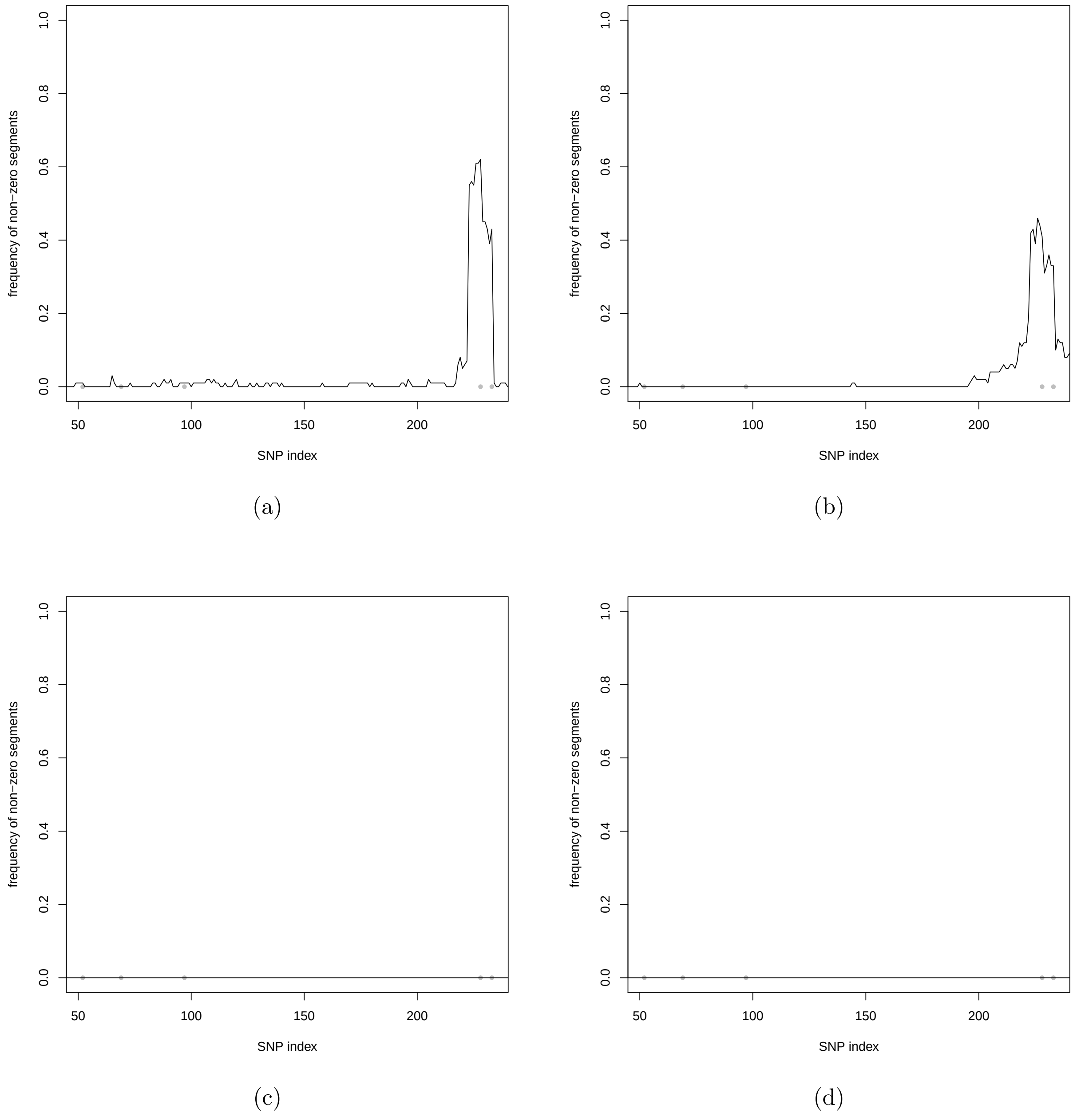

Figure D Simulation with five QTLs, $n = 100$ and 100 repetitions. Detection of non-zero segment effects using the (a) uncorrelated prior P1, (b) correlated prior P2, (c) adaptive prior P3, (d) adaptive prior P4. Gray dots indicate the simulated QTL positions.

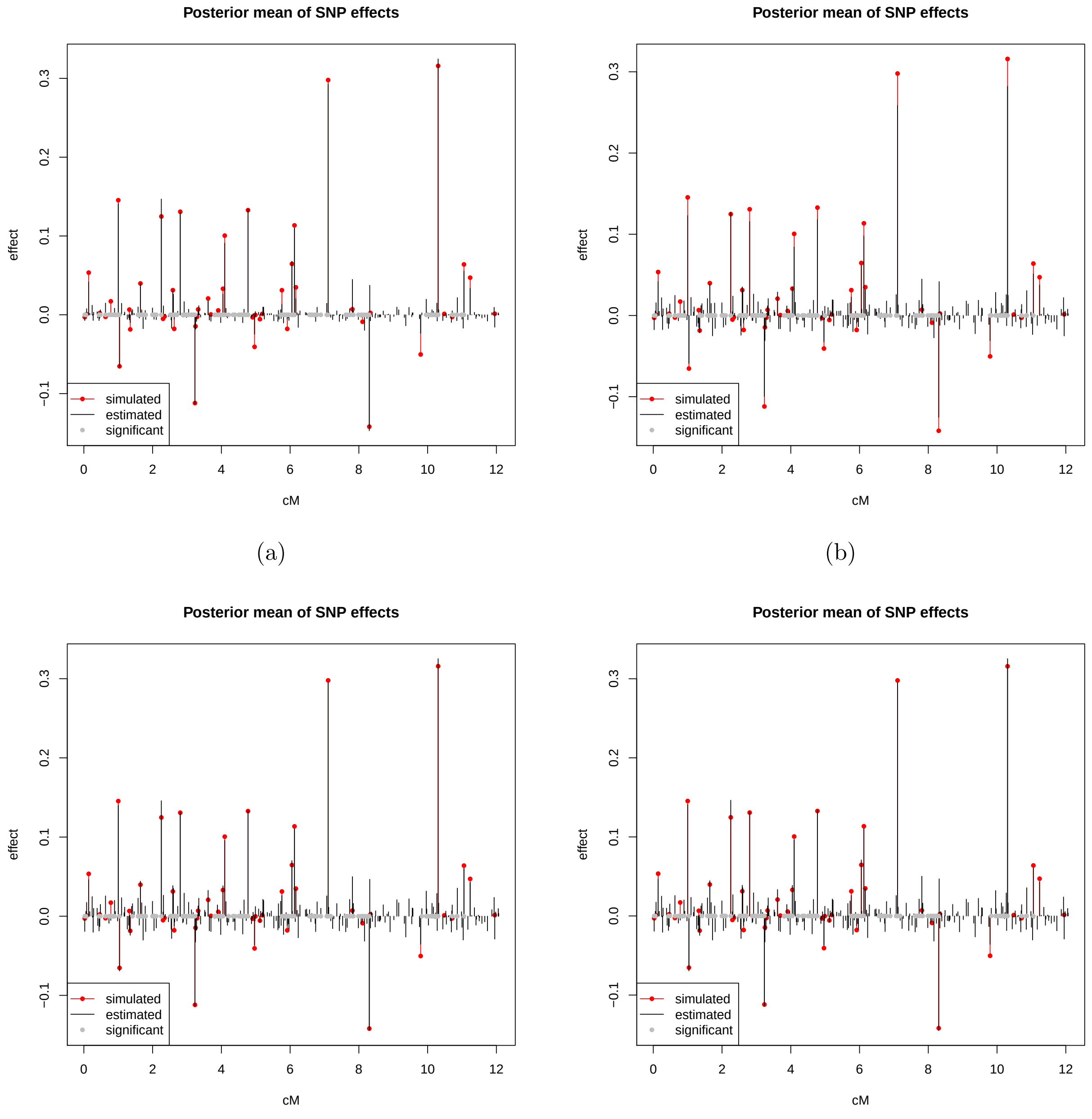

(a) (b) (c) (d)

Figure E Simulation with 50 QTLs and $n = 10\,000$. Estimated SNP effects using the (a) uncorrelated prior P1, (b) correlated prior P2, (c) adaptive prior P3, (d) adaptive prior P4.

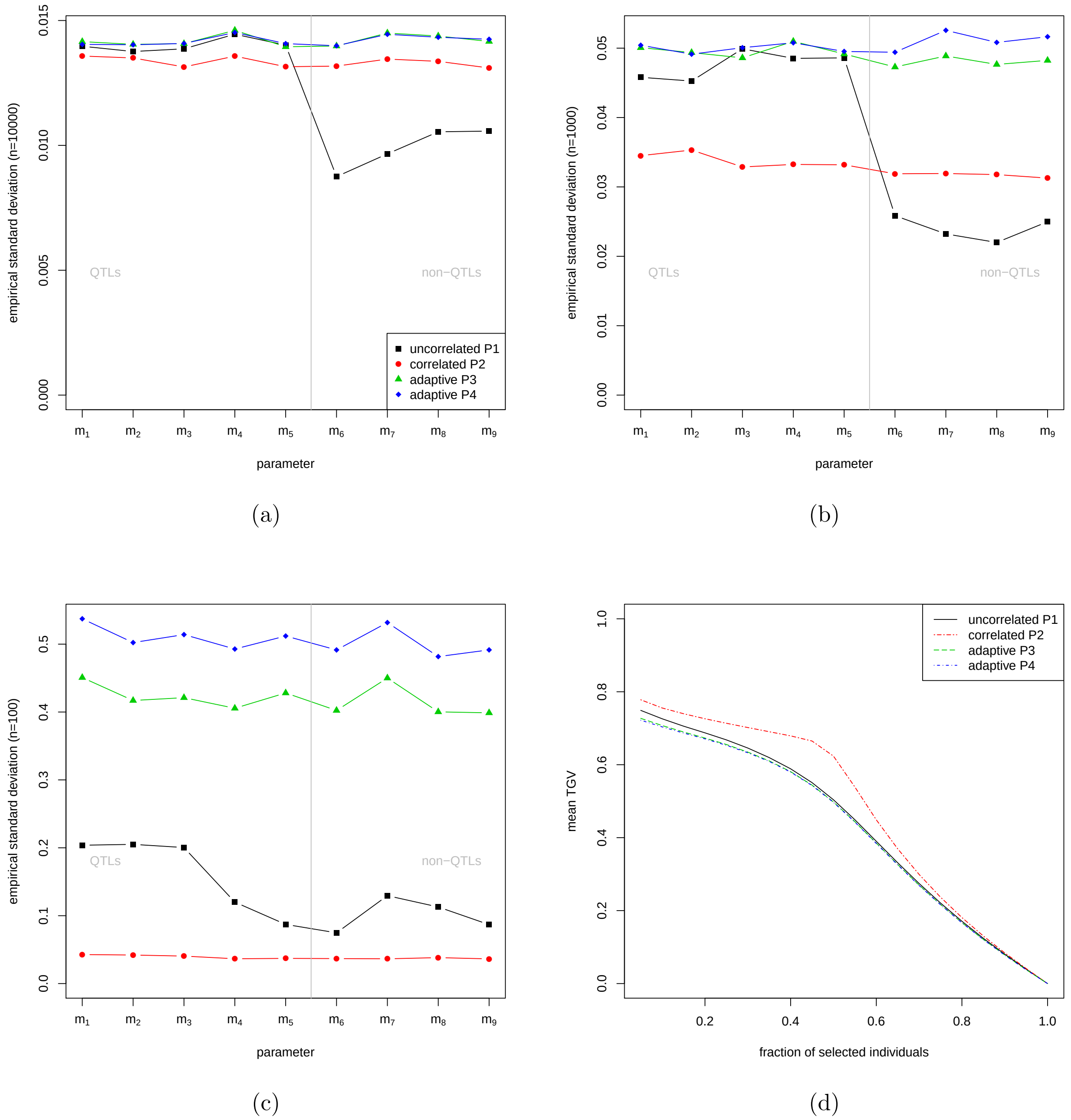

Figure F Simulation with 50 QTLs. SD of estimated effects at key SNPs for different sample sizes based on one MCMC run: (a) $n = 10\,000$, (b) $n = 1\,000$, (c) $n = 100$; (d) mean of TGV of individuals which were selected by their EGV based on 100-fold cross-validation (size of training set $n = 100$).

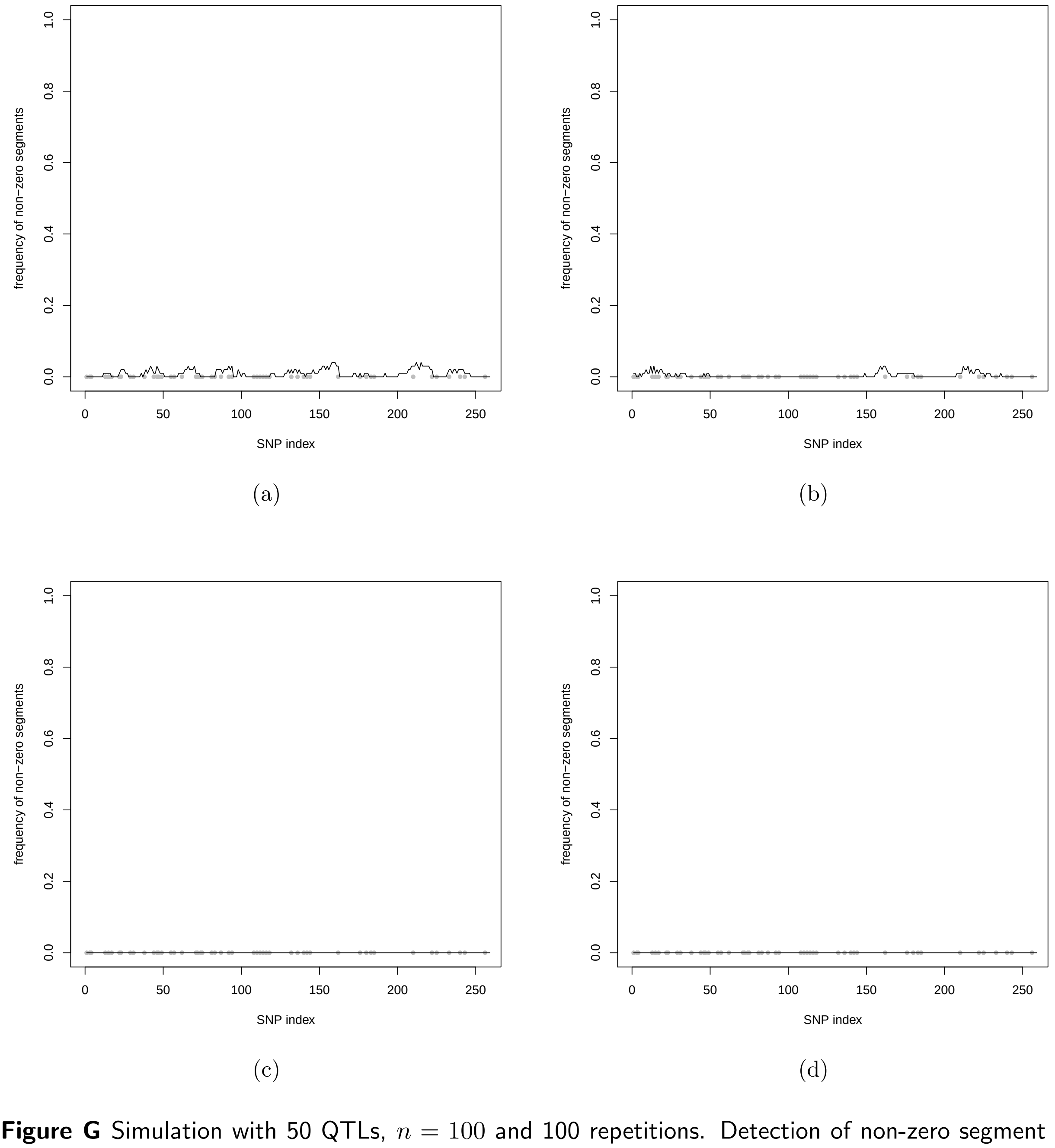

(a)

(b)

(c)

(d)

Figure G Simulation with 50 QTLs, $n = 100$ and 100 repetitions. Detection of non-zero segment effects using the (a) uncorrelated prior P1, (b) correlated prior P2, (c) adaptive prior P3, (d) adaptive prior P4. Gray dots indicate the simulated QTL positions.

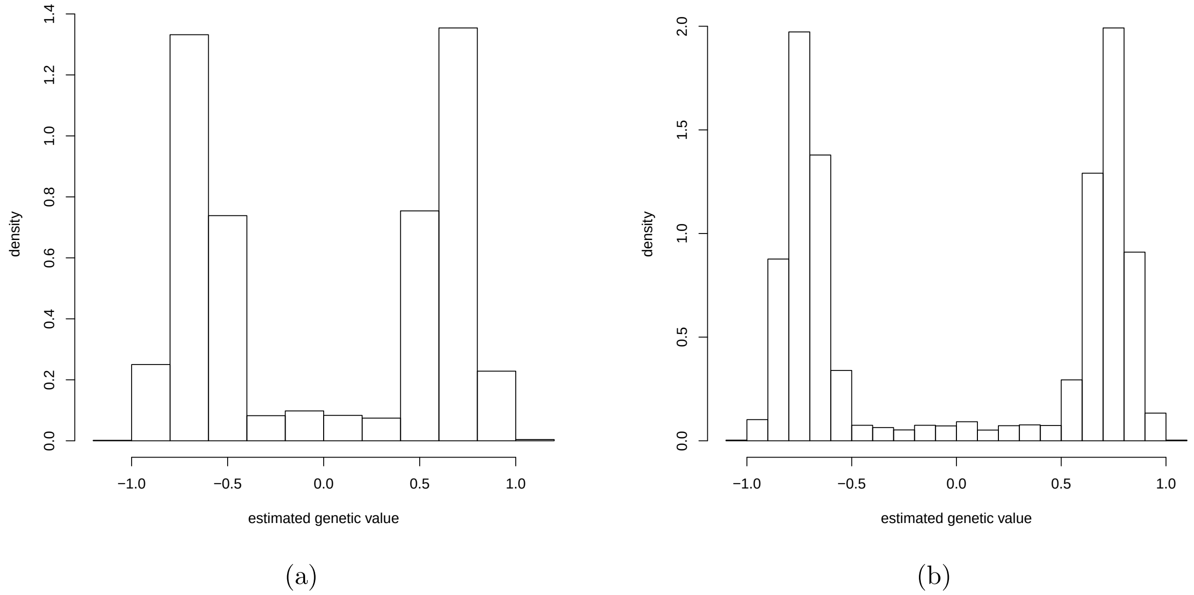

Figure H Simulation with (a) five QTLs and (b) 50 QTLs. Histogram of EGV based on estimated SNP effects using the correlated prior P2 in a single training set with $n = 100$.

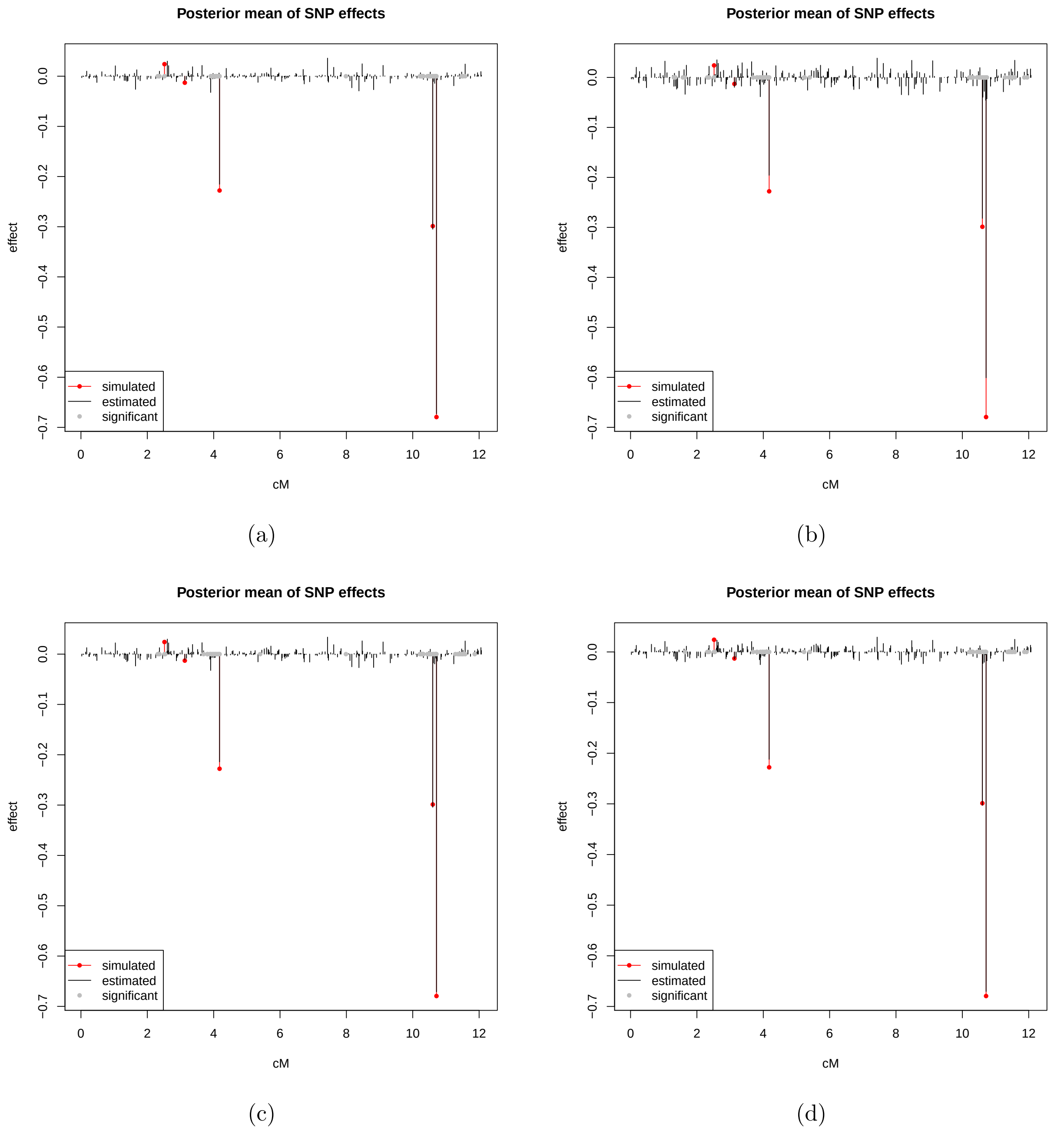

Figure I Simulation with five QTLs and $n = 10\,000$. Estimated SNP effects using the **sparse** inverse covariance matrix and (a) uncorrelated prior P1, (b) correlated prior P2, (c) adaptive prior P3, (d) adaptive prior P4.

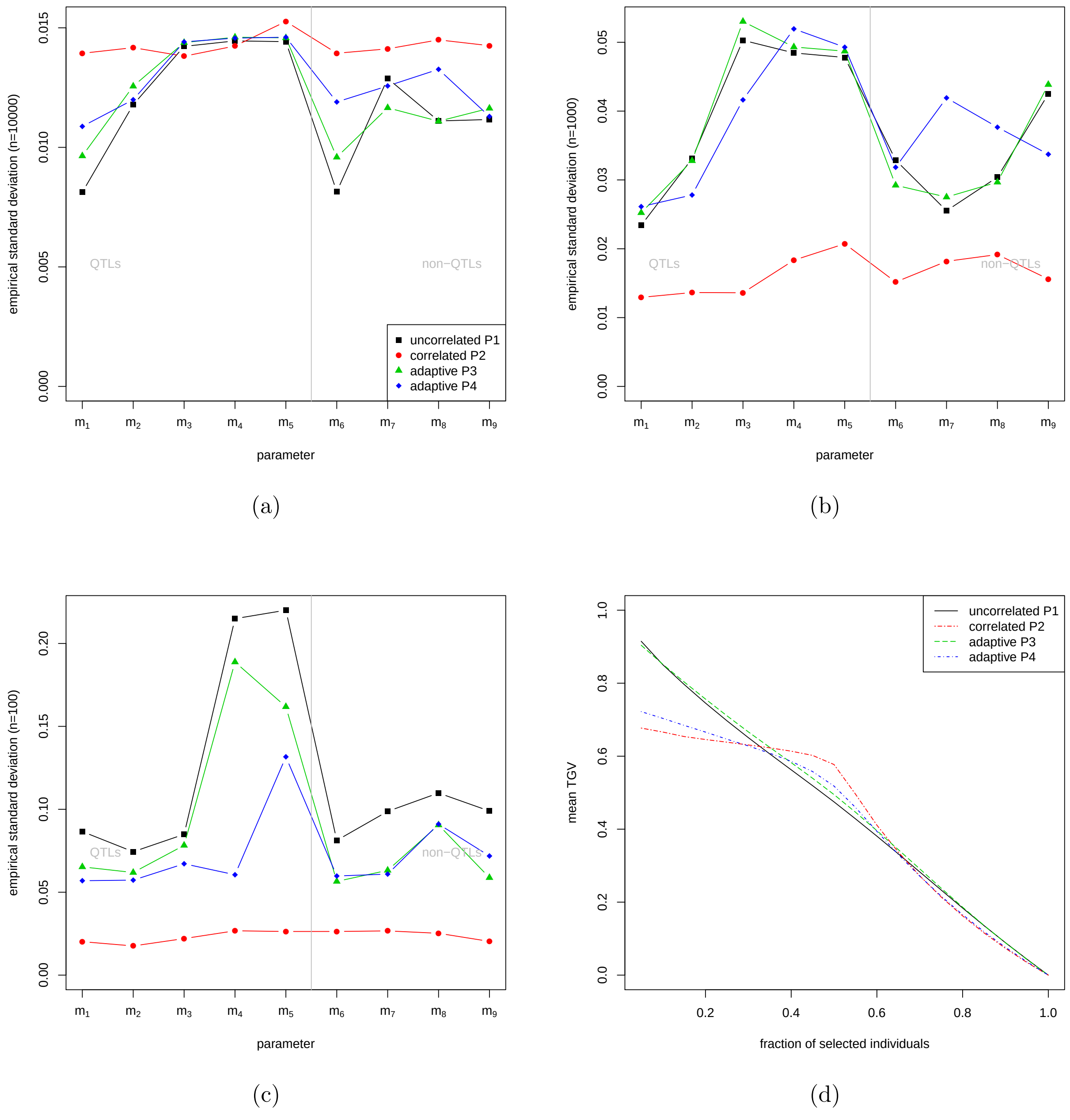

Figure J Simulation with five QTLs. SD of estimated effects at key SNPs for different sample sizes based on one MCMC run using the **sparse** inverse covariance matrix: (a) $n = 10\,000$, (b) $n = 1\,000$, (c) $n = 100$; (d) mean of TGV of individuals which were selected by their EGV based on 100-fold cross-validation (size of training set $n = 100$).

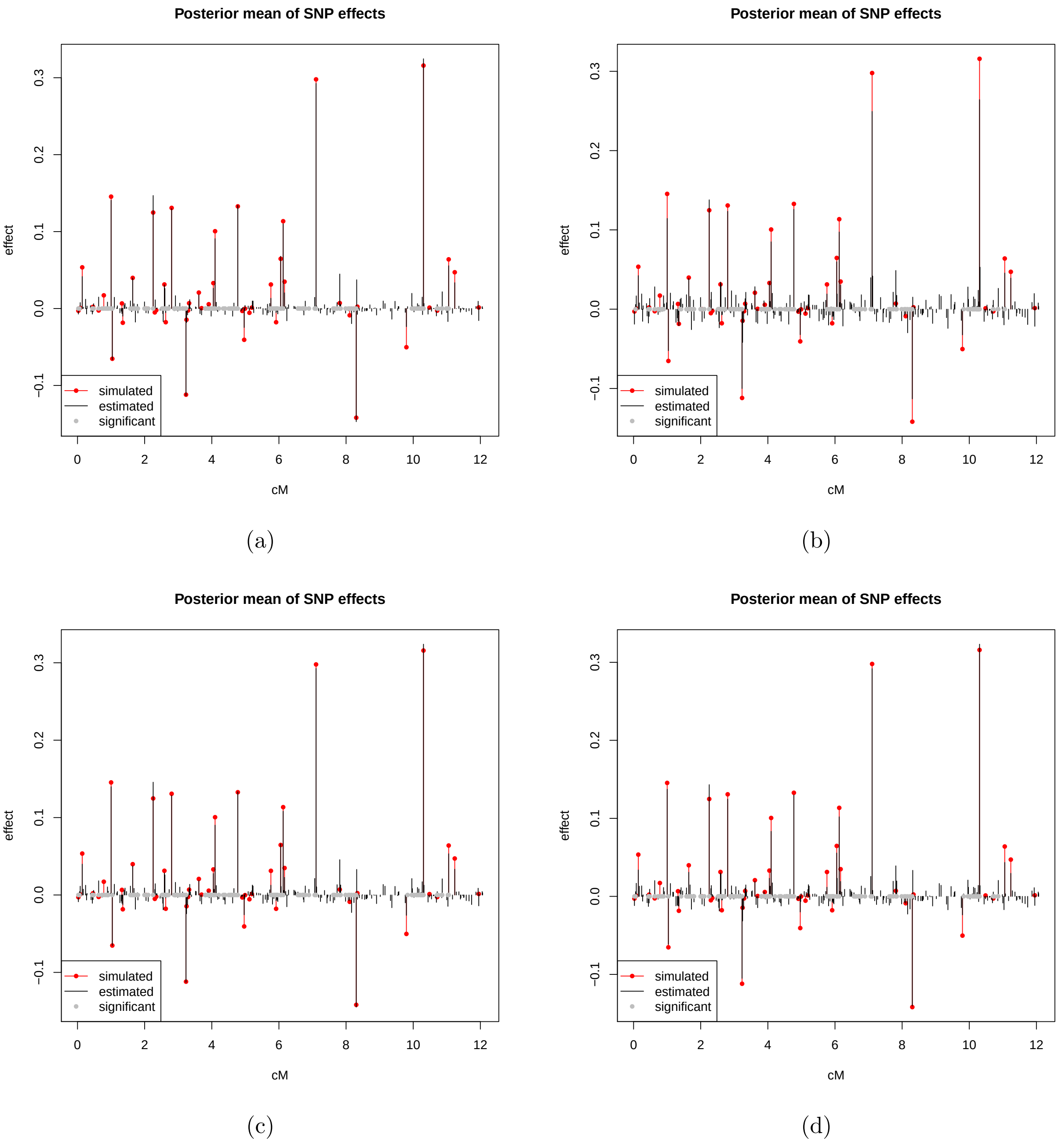

Figure K Simulation with 50 QTLs and $n = 10\,000$. Estimated SNP effects using the **sparse** inverse covariance matrix and (a) uncorrelated prior P1, (b) correlated prior P2, (c) adaptive prior P3, (d) adaptive prior P4.

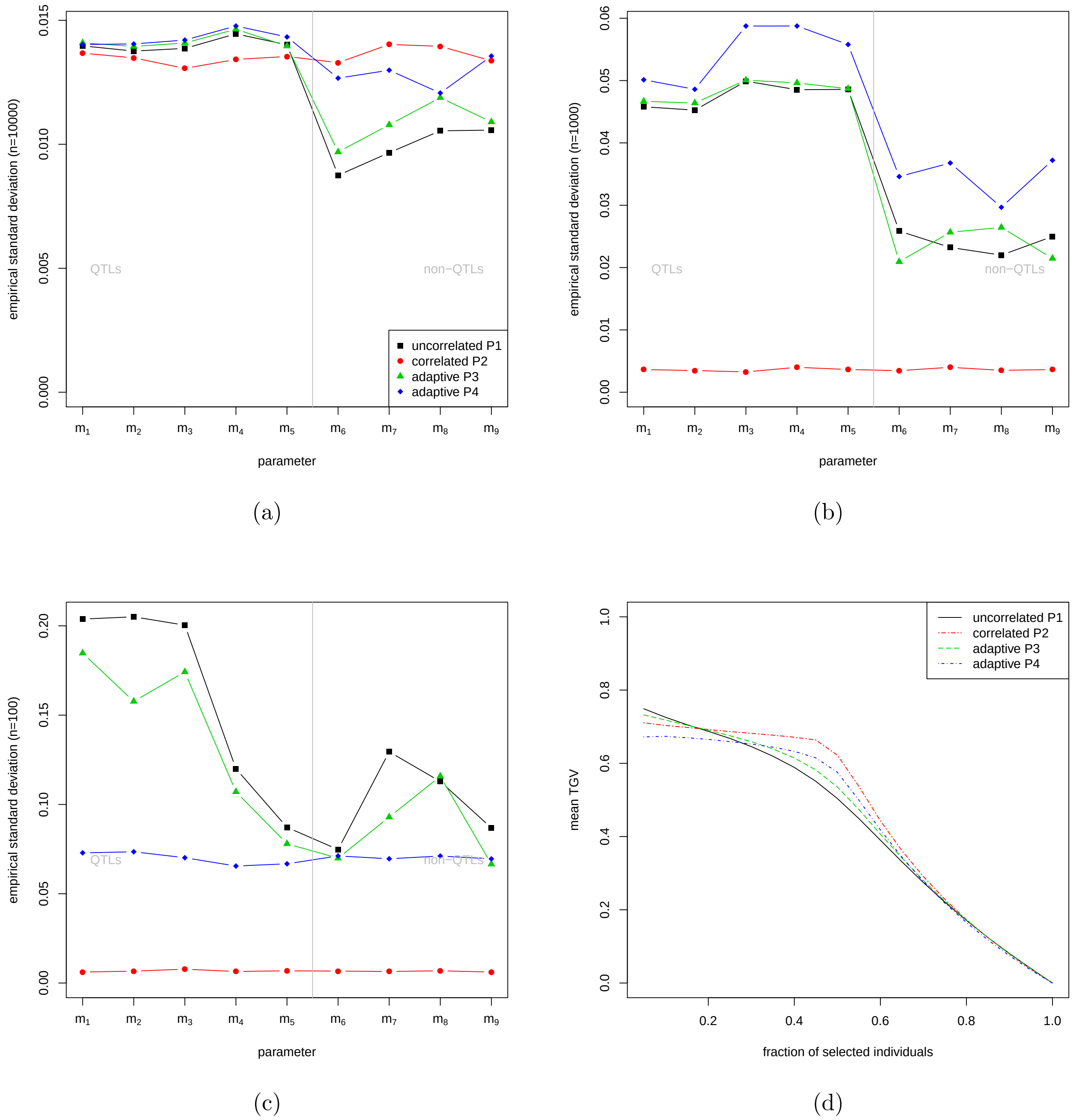

Figure L Simulation with 50 QTLs. SD of estimated effects at key SNPs for different sample sizes based on one MCMC run using the **sparse** inverse covariance matrix: (a) $n = 10\,000$, (b) $n = 1\,000$, (c) $n = 100$; (d) mean of TGV of individuals which were selected by their EGV based on 100-fold cross-validation (size of training set $n = 100$).

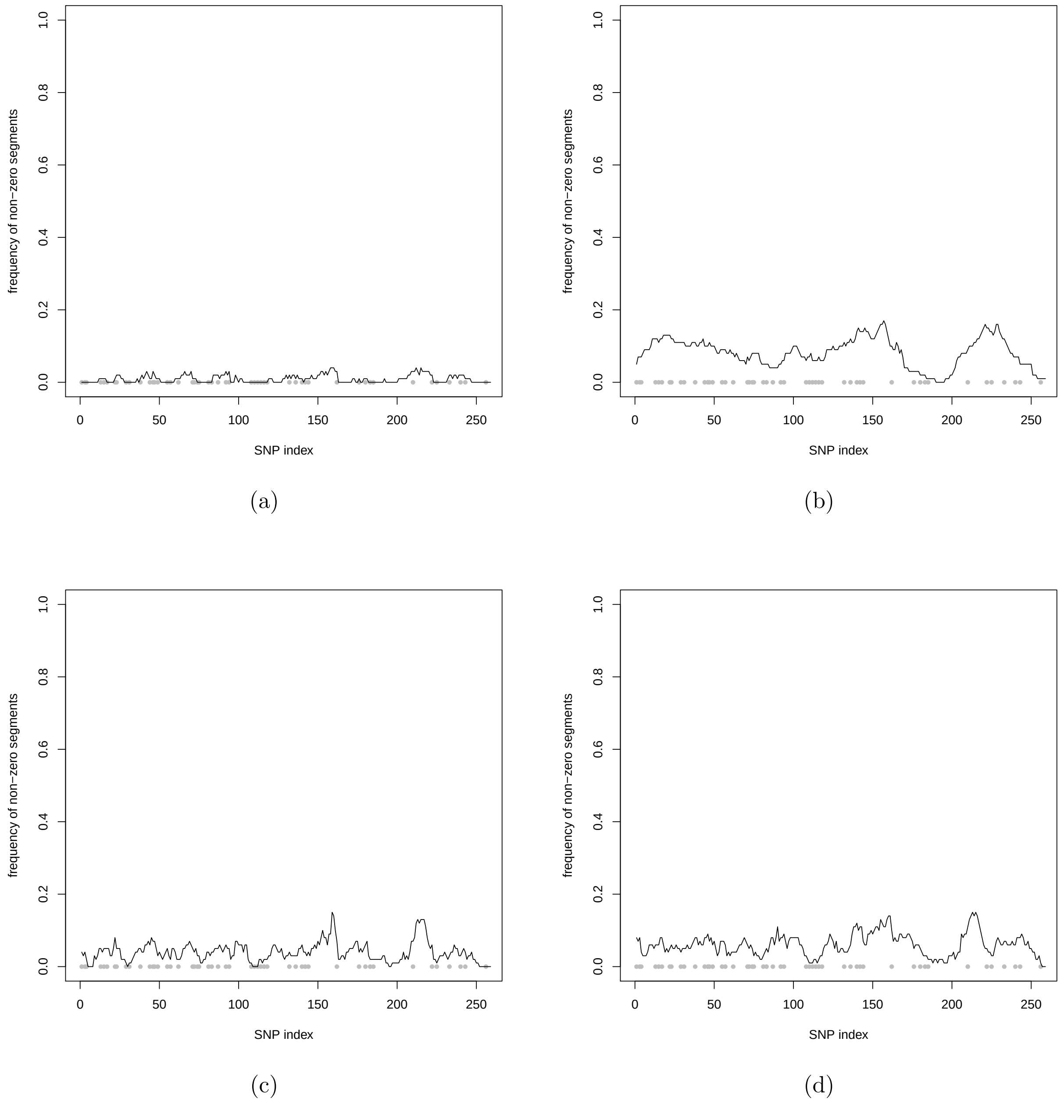

Figure M Simulation with 50 QTLs, $n = 100$ and 100 repetitions. Detection of non-zero segment effects using the **sparse** inverse covariance matrix and (a) uncorrelated prior P1, (b) correlated prior P2, (c) adaptive prior P3, (d) adaptive prior P4. Gray dots indicate simulated the QTL positions.

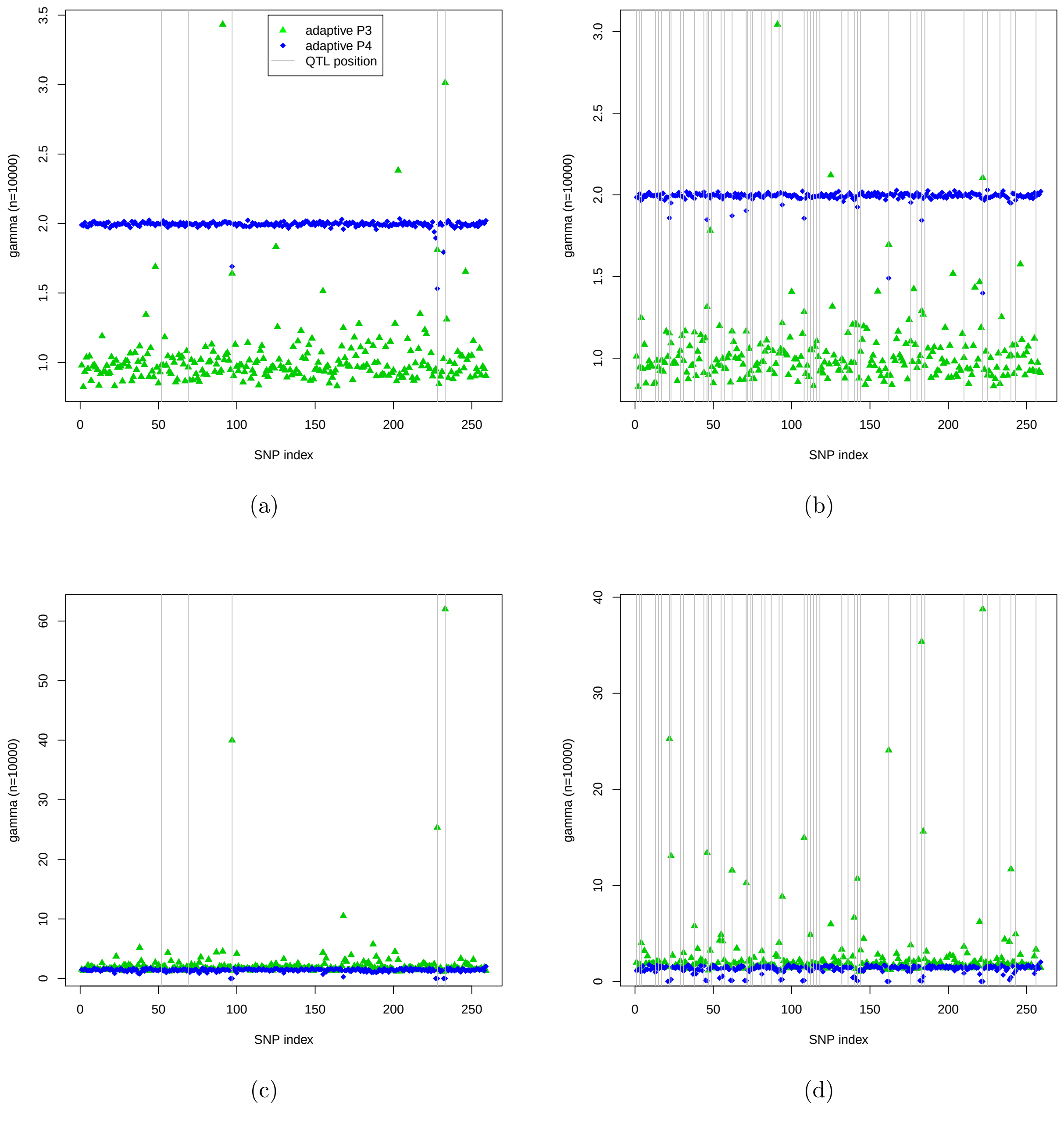

Figure N Estimated regularization parameter γ at each SNP position with priors P3 and P4 based on simulated data and $n = 10\,000$: (a) five QTLs, (b) 50 QTLs, (c) five QTLs and sparse inverse covariance matrix, (d) 50 QTLs and sparse inverse covariance matrix.

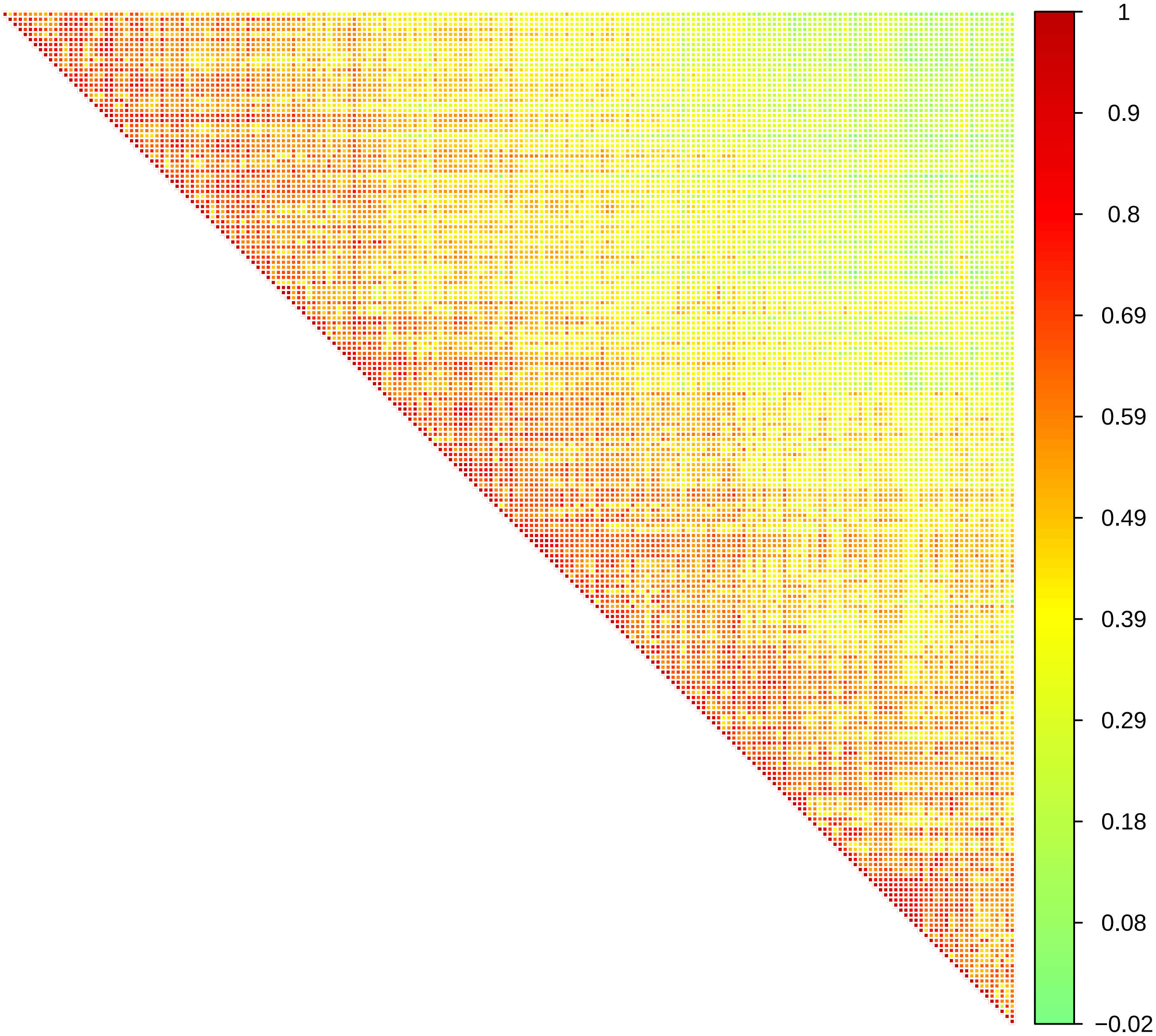

Figure O Theoretical correlation matrix of SNP 1–200 on BTA1 for the real genotypes (before bending).

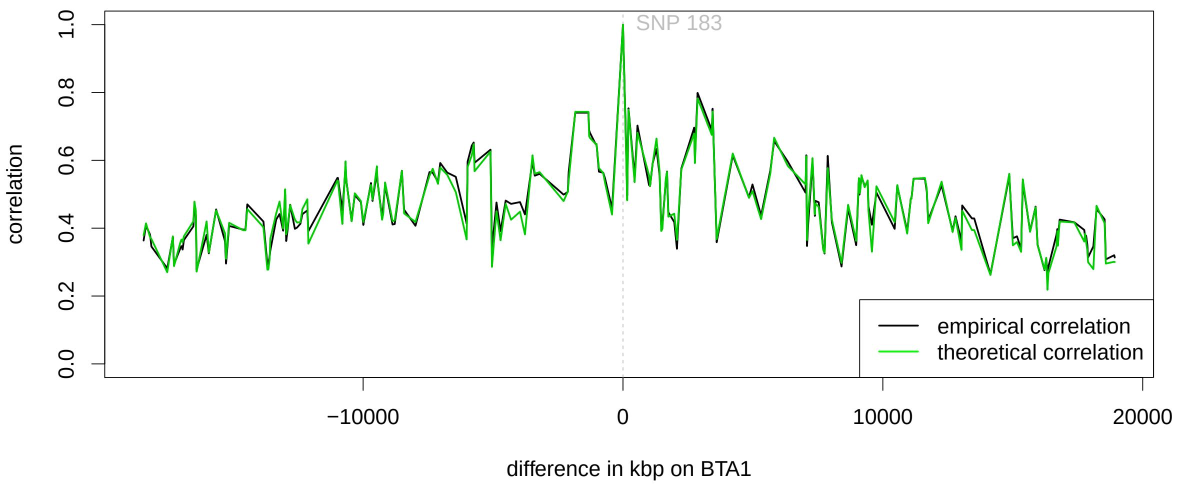

Figure P Theoretical versus empirical correlation for a randomly selected SNP based on the real genotypes.

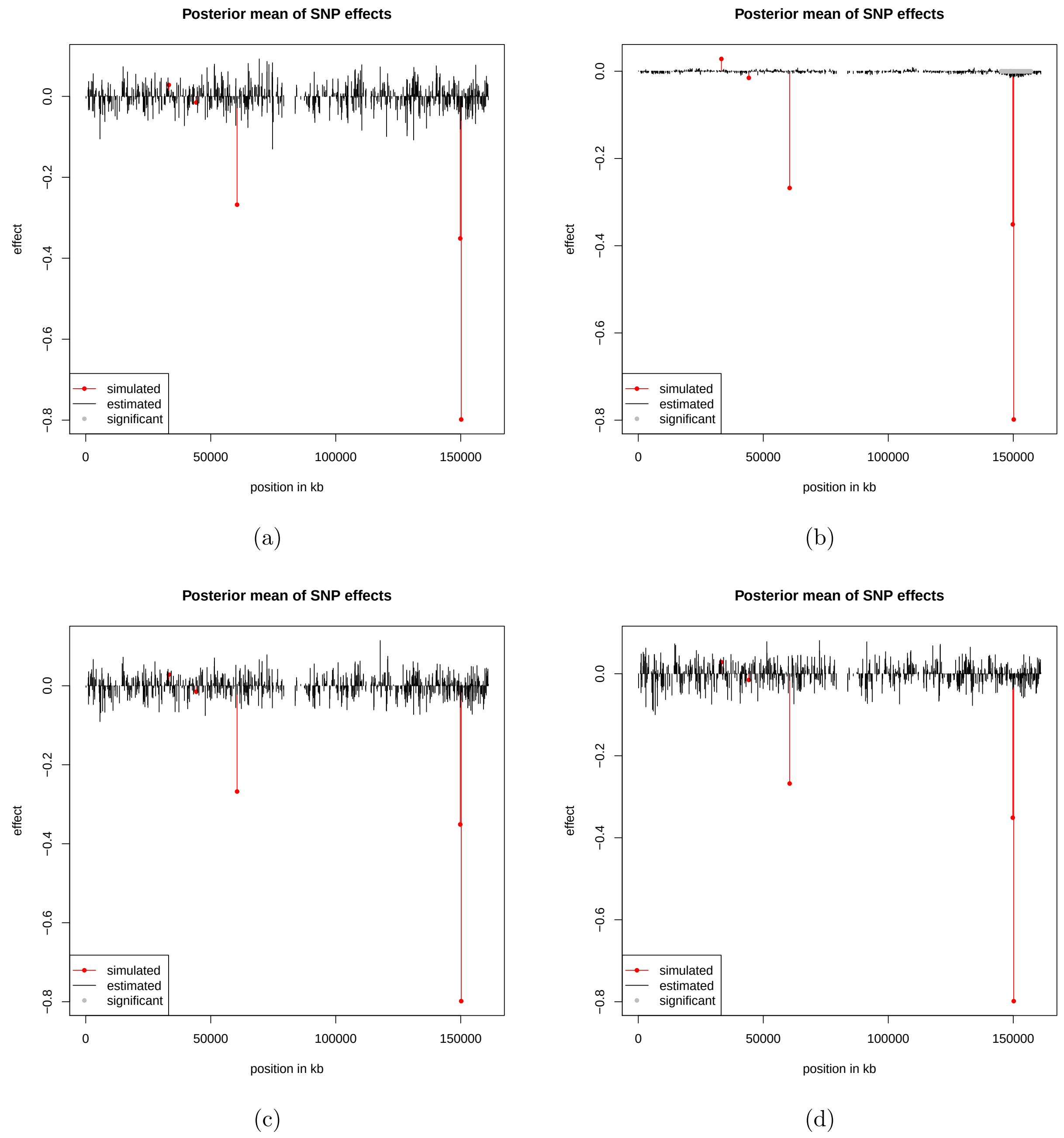

Figure Q Semi-real data with five QTLs and $n = 106$. Estimated SNP effects using the (a) uncorrelated prior P1, (b) correlated prior P2, (c) adaptive prior P3, (d) adaptive prior P4.
